# Quantitative comparison of flowering phenology traits among trees, perennial herbs, and annuals in a temperate plant community

**DOI:** 10.1002/ajb2.1387

**Published:** 2019-11-14

**Authors:** Ai Nagahama, Tetsukazu Yahara

**Affiliations:** ^1^ Graduate School of Systems Life Sciences Kyushu University Fukuoka 819‐0395 Japan; ^2^ Kyushu Open University Fukuoka 819-0395 Japan

**Keywords:** life form, pollinator attraction, reproductive assurance, resource availability, interspecific comparison

## Abstract

**Premise:**

Flowering phenology may differ among life forms due to the costs and benefits to attract pollinators, dependence on outcross pollination, and resource availability in their habitats. However, few studies have compared flowering phenology among life forms within a community and described flowering phenology at the individual, species, and community levels.

**Methods:**

We recorded flowering events for individuals of insect‐pollinated trees, perennial herbs, and annuals from spring to summer of 2016 and 2017 in a warm‐temperate forest in Japan. To compare phenological variables including mean and variance of flowering length, we standardized the number of observed individuals for each species and tested differences in variables, considering the phylogenetic relationships among species.

**Results:**

Total flowering length in trees (9–50 d) was significantly shorter than perennial herbs (27–113 d) or annuals (22–89 d), but mean flowering length was not significantly different among them. Flowering length variance was significantly smaller and intraspecies synchrony significantly higher in trees than in perennial herbs and annuals. At the community level, flowering times largely overlapped among successively flowering species, but interspecies synchrony was positive for all life forms.

**Conclusions:**

Shorter total flowering length and higher intraspecific synchrony in trees are explained by a modified pollinator attraction hypothesis suggesting that selection favors higher intraspecific synchrony because it promotes between‐individual movement of pollinators. At the community level, positive interspecific synchrony for all life forms supports the hypothesis that flowering times tend to converge among species.

In angiosperms, flowering phenology varies widely among species. This interspecific variation in flowering phenology has been examined in a range of tropical and temperate forests. Initial comparative studies on trees in tropical forests distinguished two classes of flowering phenology (Janzen, 1971): mass flowering, in which individuals flower synchronously with short durations (Heinrich and Raven, 1972; Augspurger, 1983; Bawa, 1983; seasonal flowering by Frankie et al., 1974; big bang by Gentry, 1974) and extended flowering, in which individuals flower less synchronously with long durations (Frankie et al., 1974; Bawa, 1983; steady‐state flowering by Gentry, 1974; Augspurger, 1983). According to Frankie et al. (1974), extended flowering is common in nonseasonal environments such as tropical rain forests. Later, Rathcke and Lacey (1985) reviewed the studies on tropical rain forests and concluded that mass flowering was common among trees that flower during the dry season, whereas extended flowering was found for most understory species. These two flowering patterns were also observed in temperate forests, where mass flowering was common for canopy trees and extended flowering was found for understory species (Yumoto, 1987, 1988). Further studies on tropical rain forest trees showed that these flowering patterns were two extremes of a continuous variation, and Newstrom et al. (1994) proposed three categories of annual flowering patterns: brief flowering (<1 month), intermediate flowering (1–5 months), and extended flowering (>5 months).

While studies of flowering phenology are biased toward trees, interspecific variation in flowering phenology of herbs has also been examined since the pioneering studies of Schemske (1977) and Schemske et al. (1978). In a review of those studies, Rathcke and Lacey (1985) pointed out that large plants of annuals and of perennials including herbs and shrubs tend to produce more flowers than small plants over a longer duration and argued that flowering patterns are influenced by resource availability. Another notable finding for herbs is that the flowering phenology of herbs in tropical seasonal communities is more tightly linked to rainy seasons compared with trees (Batalha and Martins, 2004; Joshi and Janarthanam, 2004). Those studies suggest that patterns of flowering phenology may differ between trees and herbs within a community, reflecting differences in resource availability and in responses to climatic factors. However, few comparisons were made for patterns of flowering phenology between trees and herbs within a community, and those comparisons have been confined to tropical seasonal communities (Batalha and Martins, 2004; Joshi and Janarthanam, 2004; Marques et al., 2004; Cortés‐Flores et al., 2017) except one study for temperate, subalpine, and alpine vegetation in Japan (Kato et al., 1993). Thus, it is still unclear how patterns of flowering phenology differ among trees, perennial herbs, and annuals within and among various plant communities. To fill this gap, we compared patterns of flowering phenology among trees, perennial herbs, and annuals in a plant community in a temperate climate using the following quantitative variables.

Flowering phenology is quantitatively defined as a time‐series distribution of the number of flowers characterized by variables such as onset date, mean, variance, and skewness of flowering length, and synchrony of flowering among individuals (Rathcke and Lacey, 1985). However, these variables were not always fully described in previous studies. To deepen our understanding of the variability of flowering phenology and its adaptive significance, we need to compare the above quantitative variables of flowering phenology among various species. In particular, we need to distinguish mean flowering length from total flowering length because total flowering length is determined not only by mean flowering length but also by variance of flowering length. This distinction is important when considering the adaptive significance of flowering phenology because mean and variance of flowering length may evolve independently under different selection pressures. Synchrony of flowering among individuals is another key variable of flowering phenology. It can be measured by indicators describing the temporal distribution of flowering individuals including the aggregation index Morisita's *Iδ* (Morisita, 1959; Yumto, 1987), the variance of onset day, or the variance of flowering length. These quantitative variables can help explain the adaptive significance of flowering patterns.

Various hypotheses have been proposed to explain the adaptive significance of flowering patterns, and these can be summarized as follows (Rathke and Lacey, 1985). First, individuals that bloom synchronously for short durations can attract many generalist pollinators with a large floral display (Janzen, 1967; Kacelnik et al., 1986; Fenner, 1998; Ohashi and Yahara, 2002; Makino et al., 2007; Nattero et al., 2011; Cortés‐Flores et al., 2017). On the other hand, individuals that bloom less synchronously over longer durations may be advantageous for flowers pollinated by specialist pollinators that visit flowers infrequently but have high flower constancy (Heinrich et al., 1977). This hypothesis was supported by Yumoto (1987, 1988) who showed that flowers of tall trees bloomed synchronously for shorter durations and attracted many generalist pollinators in the canopy, whereas flowers of understory trees bloomed less synchronously for longer durations and attracted more specialized pollinators. Hereafter, we call this the pollinator attraction hypothesis. Second, individuals of self‐incompatible species are expected to flower longer than self‐compatible plants (Pojar, 1974) because more opportunities for pollination would be expected by flowering longer (Primack, 1985; Rathcke and Lacey, 1985). In self‐incompatible individuals in which pollen transfer by pollinators is obligatory for reproduction, longer flowering durations are considered advantageous for ensuring pollination success under uncertain pollinator activity due to daily fluctuations in weather conditions (Schemske and Lande, 1985; Yumoto, 1986) or between‐year climate change (Primack, 1985; Rathcke and Lacey, 1985). Hereafter, we call this hypothesis the pollination insurance hypothesis. Third, individuals with larger plant size can accumulate more resources and flower longer because the number of flowers is known to increase with plant size (Samson and Werk, 1986; Bazzaz et al., 1987; Fabbro and Korner, 2004) and the flowering length of individuals increases with the number of flowers in trees (Otárola et al., 2013), perennial herbs, and annuals (Rathcke and Lacey, 1985; Ollerton and Lack, 1998). Also, plants (typically annuals) growing in unpredictable habitats flower earlier and longer to ensure seed production before dying due to disturbance (Rathcke and Lacey, 1985). Hereafter, we call this the resource availability hypothesis.

To determine which hypothesis better fits our data for a temperate plant community, we derived the following predictions for trees, perennial herbs, and annuals of insect‐pollinated species. We excluded wind‐pollinated species from our study because pollinator attraction and pollination insurance hypotheses hold only for insect‐pollinated species. At our study site, the trees we observed are pollinated by generalist insect pollinators including hymenopterans, dipterans, lepidopterans, and coleopterans (Kuwata, 2013; Appendix S1). According to the pollinator attraction hypothesis (Table 1, top), tree individuals are predicted to flower for a shorter duration and more synchronously to attract generalist pollinators (Yumoto, 1987, 1988). On the other hand, individuals of perennial herbs specialized for particular pollinators such as bees are predicted to flower longer and less synchronously (Yumoto, 1987, 1988). In our study, the latter prediction is the case for nonweedy perennial herbs (Appendix S2). For weedy perennial and annual herbs, individuals are predicted to bloom less synchronously because those species are often selfing or asexually reproducing. The pollinator attraction hypothesis does not lead to any specific predictions about the flowering period of weedy herbs. According to the pollination insurance hypothesis (Table 1, middle), tree individuals, which are highly outcrossing, are predicted to flower longer, and individuals of annual herbs are predicted to flower for a shorter duration (Pojar, 1974; Primack, 1985; Abe, 2001). Perennial herbs are predicted to flower longer than annual herbs because they include more outcrossing species than annual herbs (Appendix S2; see also Baker, 1974). According to the resource availability hypothesis (Table 1, bottom), flowering period is expected to increase as plant size increases for the categories of annuals, perennial herbs, and trees, if habitats are predictable. We can also predict that variance of flowering length increases with plant size, because variation in the amount of available resource (e.g., nitrogen) among individuals will be larger in larger plants. This prediction means that trees show larger variance of flowering length than perennial herbs and annuals. On the other hand, in unpredictable habitats, annuals should flower earlier and longer, with larger variance, to insure some seed production even when the growing season is cut short (Rathcke and Lacey, 1985). These predictions can be examined using quantitative variables such as onset date, mean, variance, and skewness of flowering length, and synchrony of flowering among individuals.

**Table 1:** Predictions based on three hypotheses for trees, perennial herbs, and annuals.

Hypothesis	Trees	Perennial herbs	Annuals
Pollinator attraction	Individuals flower for a shorter period and higher synchronously than herbs.	In nonweedy (more outcrossing) species adapted to specialized pollinators, individuals flower longer and less synchrously than trees.	Individuals flower less synchronously than trees; no specific prediction for flowering period.
		In weedy (selfing) species, individuals flower less synchronously; no specific prediction for flowering period.	
Pollination insurance	Individuals flower longer than perennial herbs.	Individuals flower for shorter periods than trees and longer than annuals.	Individuals flower for shorter periods than perennial herbs.
Resource availability	Tree individuals flower longer than herbs, with larger variance.	Flowering length and its variance intermediate between trees and annuals.	In predictable habitats, annuals smaller than perennials flower shorter, with smaller variance. In unpredictable habitats, annuals flower earlier and longer, with larger variance.
			In unpredictable habitats, annuals flower earlier and longer, with larger variance.

On the basis of the data obtained for this study, we could also compare flowering phenology between species. Some have claimed that plant species may evolve traits that decrease phenological overlap with other species competing to attract common pollinators (van Schaik et al., 1993). However, Rathcke and Lacey (1985) reviewed empirical studies and concluded that interspecific divergence in flowering within plant communities is rarely supported by statistical tests. Yumoto (1987, 1988) suggested that flowering phenology among canopy species is asynchronous to avoid competition for generalist pollinators. In addition, Sakai et al. (1999) suggested that plant species that attract specialist pollinators flower synchronously with other species. The ideas of Yumoto (1987, 1988) and Sakai et al. (1999) support the hypothesis that flowering phenology is more synchronous among herbaceous species than among trees.

We address the following specific questions regarding patterns of flowering phenology in a temperate plant community. (1) Do intraspecific measures of flowering phenology variables, including total, mean, and variance of flowering length, differ among trees, perennial herbs, and annuals? (2) Which predictions of the pollinator attraction, pollination insurance, and resource availability hypotheses better explain our observations on intraspecific patterns of flowering phenology? (3) Does interspecific synchrony of flowering phenology differ among trees, perennial herbs, and annuals?

## MATERIALS AND METHODS

### Observations

**Figure 1:**
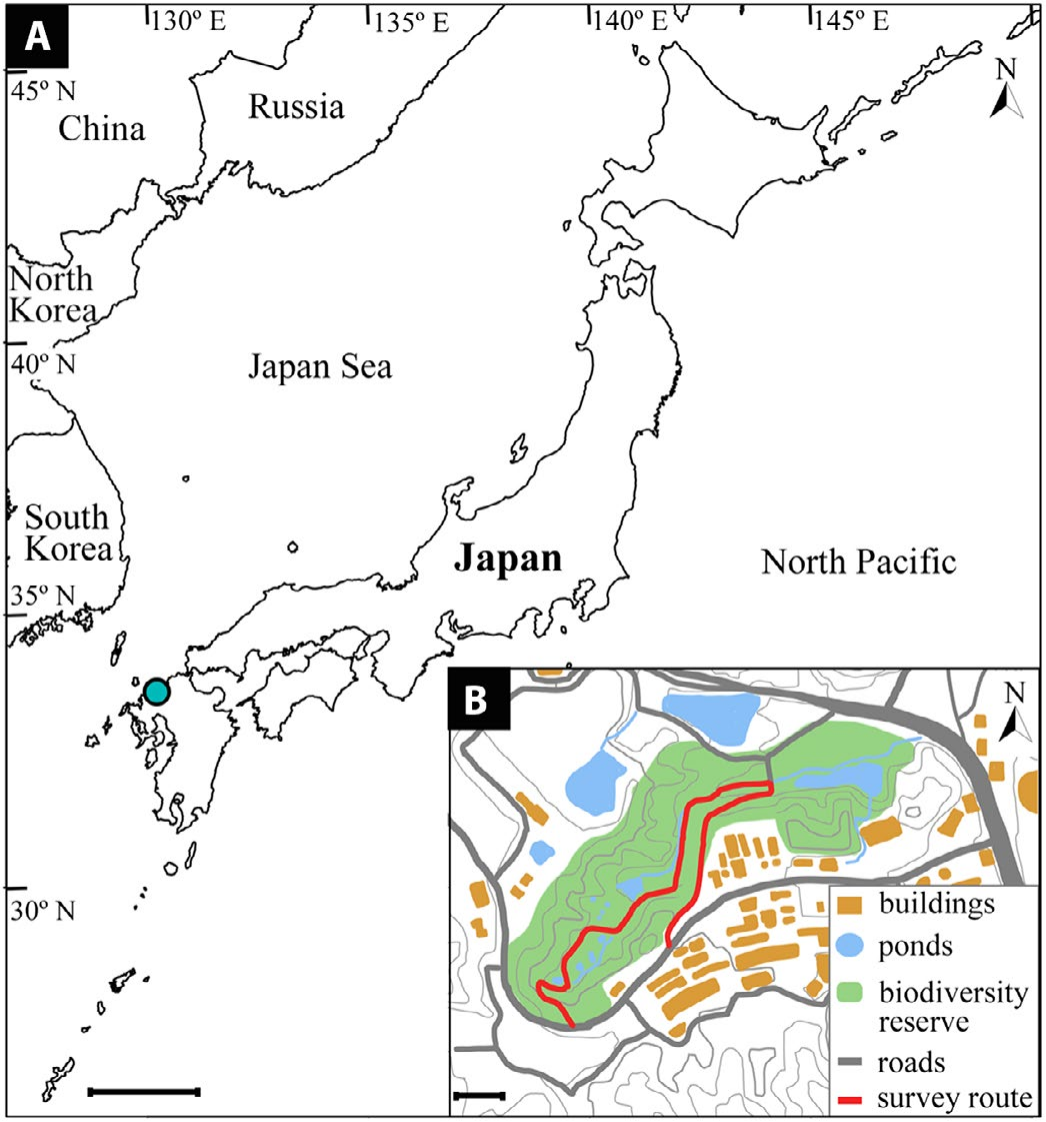
Map of study site and survey route. (A) Blue dot marks Fukuoka, Japan. Scale bar = 200 km. (B) Close up of study site showing survey route. Scale bar = 100 m.

Plants were monitored for flowering once a week from 1 March to 31 July in 2016 and 2017 in the biodiversity reserve of Ito campus (33°35′47.5′′N, 130°12′50.0′′E; Fig. 1), Kyushu University, Fukuoka, Japan, an area of about 37 ha at an elevation from 20 to 57 m a.s.l., where monthly average temperatures fluctuated from 6.2°C in January to 27.4°C in August (a 21.2°C difference), and monthly precipitation fluctuated from 75.5 mm in March to 337 mm in June (261.5 mm difference) (Kyushu University 2018; Appendix S3). The reserve is located in a small valley facing northeast, surrounded by two ridges running from southwest to northeast that are covered with evergreen broad‐leaved forest dominated by *Quercus glauca* Thunb.*, Castanopsis sieboldii* (Makino) Hatus., and *Neolitsea sericea* (Blume) Koidz. mixed with some deciduous trees including *Mallotus japonicus* (L.f.) Müll.Arg., *Celtis sinensis* Pers., and *Aphananthe aspera* Planch. The central area of the reserve lies between a small stream and a road and is maintained as an open grassland by mowing; three small ponds are surrounded by tall grass. A forest margin along the road is covered with herbaceous vegetation composed of weedy annuals such as *Galium spurium* L. and *Corydalis incisa* Pers. and perennial herbs such as *Trifolium repens* L. and *Farfugium japonicum* (L.) Kitam. Approximately 650 plant species have been recorded in the biodiversity reserve. Among them, we observed 48 insect‐pollinated plant species belonging to 36 genera of 24 families, which flowered for more than two observation days along the survey route (Fig. 1). Each genus included up to three species, and each family included up to four genera. Our sample included 13 outcrossing species of trees, 15 perennial herbs including 12 outcrossing species and one each of selfing, agamospermus, and vegetatively reproducing species, and 20 annuals including nine outcrossing, 10 selfing, and one agamospermus species (Appendix S2). Perennial herbs included four species of arable weeds, six species of roadside weeds, and five nonweedy species; all were polycarpic. Annuals included 17 species of arable weeds, two species of roadside weeds, and one nonweedy species (Asai, 2012, 2016).

For trees, Kuwata (2013) recorded insect flower visitors by taking a photograph every 10 min using programmable digital cameras (PENTAX Optio‐W80 and WG‐1, Ricoh Imaging Co., Tokyo, Japan) from 6 April to 30 September 2015. This method often fails to record the visits of bees including *Bombus ardens* Smith, *Xylocopa appendiculata* Smith, and *Apis mellifera* L. To record the visits of bees, we directly observed visitors to tree flowers by collecting insects on the flowers 3 h or longer per day for a total of 14 days in April, May, and June in 2018 (Appendix S1). For herbaceous species, we recorded insect flower visitors including bees by direct observation. For *Cirsium japonicum* DC., we also used the camera to record flower visitors (Appendix S1).

For trees, flowering was observed and recorded for each individual found along the survey route. For perennial herbs and annuals, flowering was observed and recorded in 50 plots of 1 × 1 m along the survey route. These small plots corresponded to one individual for large herbaceous species and multiple individuals for small herbaceous species, but for practical purposes we regarded each flowering shoot of a species in a plot as an individual because the individual small herbaceous plants in a plot were often difficult to distinguish. For each “individual”, we recorded the following dates: (1) the onset date of flowering, defined as the day when the first flower opened, and (2) the end date of flowering, defined as the day when the petals of the last flower became discolored or fell off. Flowering length of an individual was determined as the time between the onset and end days for the individual, and the total flowering length of a species was determined as the time from the onset day of the first‐flowering individual to the end day of the last‐flowering individual. The mean flowering length was determined as the arithmetic mean of flowering durations recorded for individuals of the same species.

### Phenological variables used for each species

We calculated the following phenological variables for species with five or more individuals in the study area. As a set of basic quantitative variables, we compared total flowering length of species (TFL), mean flowering length of individuals (MFL) and its variance (VFL), and skewness and kurtosis of the flowering length distribution among individuals. To display the distributions of TFL for trees, perennial herbs, and annuals, we drew violin plots using the R package ggplot2 (v.2.2.1; Wickham, 2010). The VFL describes the variation in flowering length among individuals. Skewness provides a measure of the asymmetry of the flowering length distribution; the more skewed the distribution, the more individuals flower for short durations, usually immediately after the onset day, and the fewer individuals flower near the end day. Kurtosis represents the deviation from the normal distribution and describes the weight of the distribution tail. In addition, we examined flowering synchrony among individuals using the following two measures: (1) the variance of the onset date and (2) the Morisita aggregation index (*Iδ*) (Morisita, 1959). To calculate the variance of the onset date, we standardized the onset day of the first‐blooming individual as 0. Smaller variance in the onset date represents higher synchrony among individuals. On the other hand, larger *Iδ* values represent higher synchrony among individuals. *Iδ* was calculated using the R package vegan (v.2.4.4; Oksanen et al., 2017).

### Phenological variables used for interspecific comparison

We also tested whether the synchrony of flowering phenology varied among the life forms using the following two measures: (1) skewness of the onset dates of flowering and (2) an index of community‐wide synchrony (Loreau and de Mazancourt, 2008). We calculated these indices for each life form and each year. The skewness of each life form was compared by resampling the data 1000 times. The latter index was calculated using the following formula:

$$\varphi =\frac{\sigma_{x_{T}}^{2}}{{\left(\sum \sigma_{x_{i}}\right)}^{2}}\text{,}$$

where $\sigma_{x_{T}}^{2}$ is the temporal variance of the community time series *x*_T_(*t*) = ∑*x*_*i*_(*t*), and ${\left(\sum \sigma_{x_{i}}\right)}^{2}$ is the sum of the temporal standard deviation of the time series across all species. This describes the rate of increase of flowering individuals in a species relative to the increase in flowering individuals in a community (Loreau and de Mazancourt, 2008). The index approaches 1 when the flowering of two species is highly synchronous. These two phenological variables were determined for the 39 species for which we observed five or more individuals. For calculating the community‐wide synchrony index, the R package synchrony (v.0.2.3; Gouhier and Guichard, 2014) was used.

### Construction of a phylogenetic tree and testing for phylogenetic signal

Although flowering phenology is often constrained by phylogenetic relationships among species (Davies et al., 2013; Cara‐Donna and Inouye, 2014; Du et al., 2015; Pei et al., 2015; Cortés‐Flores et al., 2017), a study showed that flowering period is not constrained by phylogenetic relationships (Cara‐Donna and Inouye, 2015). If the former is the case, we need to consider phylogenetic relationships in our analysis of the data (Felsenstein, 1985; Harvey and Pagel, 1991). If the latter is the case, we can apply standard statistical tests in which random sampling is assumed. To test which was the case for our data set, we constructed a phylogenetic tree (see below) and determined Blomberg's *K* (Blomberg et al., 2003). Blomberg's *K* compares the distribution of observed trait values with a distribution expected for trait evolution under Brownian motion (Blomberg et al., 2003; Ackerly, 2009). When *K* is 1, the observed distribution is identical to the expected distribution, indicating that the trait distribution is highly influenced by phylogenetic relationships. On the other hand, *K* values close to 0 show negligible phylogenetic signals. To test the significance of *K*, we calculated phylogenetic independent contrasts (Felsenstein, 1985) of each phenological trait value and compared them with randomly shuffled trait values across the phylogeny (Cara‐Donna and Inouye, 2015). Blomberg's *K* was determined and tested using the R package phytools (v.0.5‐10; Revell, 2012) for species with five or more individuals.

We constructed a phylogenetic tree of all observed species using DNA sequences of *rbcL* and *matK* and *Euryale ferox* Salisb. as the outgroup (Appendix S4). The DNA sequences of the observed species and the outgroup were downloaded from the National Center for Biotechnology Information (NCBI) (https://www.ncbi.nlm.nih.gov). We aligned the sequences using the program MEGA 7 (v.7.0.3). After aligning the sequences, we reconstructed phylogenetic relationships among plant species examined using the program BEAST (v. 1.8.3; Drummond et al., 2012) and the GTR (general time reversible) model (Lanave et al., 1984; Tavaré, 1986) for nucleotide substitution, gamma distribution for site heterogeneity, the lognormal relaxed clock model (Drummond et al., 2006) for lineage‐specific rate variation, the Yule process model (Yule, 1925) for diversification, and UPGMA for obtaining a tree prior. With those settings, we estimated a time‐measured phylogeny by running Markov chain Monte Carlo (MCMC) for 100 million generations, sampling every 10,000 trees, and discarding the first 1000 trees as a burn‐in. We repeated this estimation five times independently and obtained five phylogenetic trees. We obtained the maximum credibility tree from those five phylogenetic trees using the LogCombiner program of BEAST. Finally, we determined clade ages based on calibration with the ages estimated as described by Bell et al. (2010).

### Statistical analysis 1: tests of variables for each species

We observed phenological variables for 2 years (2016 and 2017), and the number of individuals observed per species varied from 1 to 69. To consider the possible effects of this variation on statistical tests of phenological variables, we used the following two methods. First, we tested whether each phenological variable (an average of a variable for a species for 2016 or 2017) varies with year and/or the number of observed individuals, using GLMM with an average of a phenological variable as the outcome variable, year and the number of individuals observed in 2016 or 2017 for each species as predictor variables, and genus as a random factor, using a lognormal link function and gamma distribution of errors. For skewness, which includes negative values, we tested the effects of year and the number of observed individuals using LMM with an identity link function and Gaussian distribution of errors including genus as a random factor. We used genus as a random factor because previous studies showed that phenological characteristics are phylogenetically constrained (Davies et al., 2013; Cara‐Donna and Inouye, 2014; Du et al., 2015; Pei et al., 2015; Cortés‐Flores et al., 2017). Thus, in addition to the test using Blomberg's *K*, we considered possible phylogenetic effects using genus as a random factor for GLMM or LMM. We used the R package lme4 (v.1.1.15; Bates et al., 2014) for these tests. For skewness, we tested differences between model 0 containing included only a random factor (genus) and the following two LMM models: model 1 containing year and a random factor (genus); model 2 containing the number of observed individuals and a random factor (genus). If model 0 significantly differed from model 1 or 2, we considered the other model to be more reliable for explaining the effects of year and the number of observed individuals on skewness. For those tests, we used 43 species, including 11 species of trees, 12 perennial herbs, and 20 annuals that had five or more observed individuals in both 2016 and 2017. If there was no significant effect of year or number of observed individuals, we further tested the differences in each phenological variable among trees, perennial herbs, and annuals using data for all species, including species with fewer than five individuals in either year. To test the difference between trees and herbs or between perennial herbs and annuals, we used a GLMM with lognormal link function and Gamma distribution of errors, in which genus was included as a random factor.

Second, to adjust the number of individuals to be compared, we determined a rarefaction–extrapolation curve for each phenological variable of each species using the following bootstrap method. We carried out bootstrapping 1000 times for each phenological variable of each species for the sample size from 1 to 69. We then fitted linear, quadratic, logarithmic, and logistic models to the relationship between a phenological variable and sample size and chose a model using the Bayesian information criterion (BIC). We conducted this model selection process for each phenological variable using data obtained by pooling bootstrap samples of all species in which the maximum of a phenological variable in each species was standardized to one. Finally, the model selected for each variable was applied to data of each species to describe the change in a phenological variable as a function of the number of observed individuals (from 1 to 69).

We tested differences in phenological variables among life forms using rarefaction–extrapolation curves as follows. First, we determined a value of each variable for 5, 7, 12, 18, or 22 individuals representing the minimum, first quartile, median, nearest integer to the average value 17.8, and third quantile in the distribution of numbers of observed individuals, respectively. Second, in each case (5, 7, 12, 18, or 22 individuals), we used *t*‐tests if data of two groups followed a normal distribution, the Wilcoxon's rank sum test if either group did not follow a normal distribution but both groups had the same variance, or the Fligner–Policello test if either group did not follow a normal distribution and the variance of the two groups differed, using R (v.3.4.1; R Core Team, 2017). In these comparisons, *p*‐values were adjusted using the Holm–Bonferroni method for multiple comparisons (Holm, 1979).

### Statistical analysis 2: tests of variables for interspecies comparison

We tested the significance of the skewness of onset date using D'Agostino's *K*‐squared test. For this calculation, we used the R package moments (v.0.14; Komsta and Novomestky, 2015). For testing the differences in the community‐wide synchrony index (Loreau and de Mazancourt, 2008) among life forms, we computed its distribution for trees, perennial herbs, and annuals using 1000 bootstraps of 11 species of trees, 12 perennial herbs, and 20 annuals, from the original data, allowing resampling of the same species. We calculated their 95% confidence intervals using R (v.3.4.1; R Core Team, 2017) and compared them among life forms.

## RESULTS

### Phenological observations

**Figure 2:**
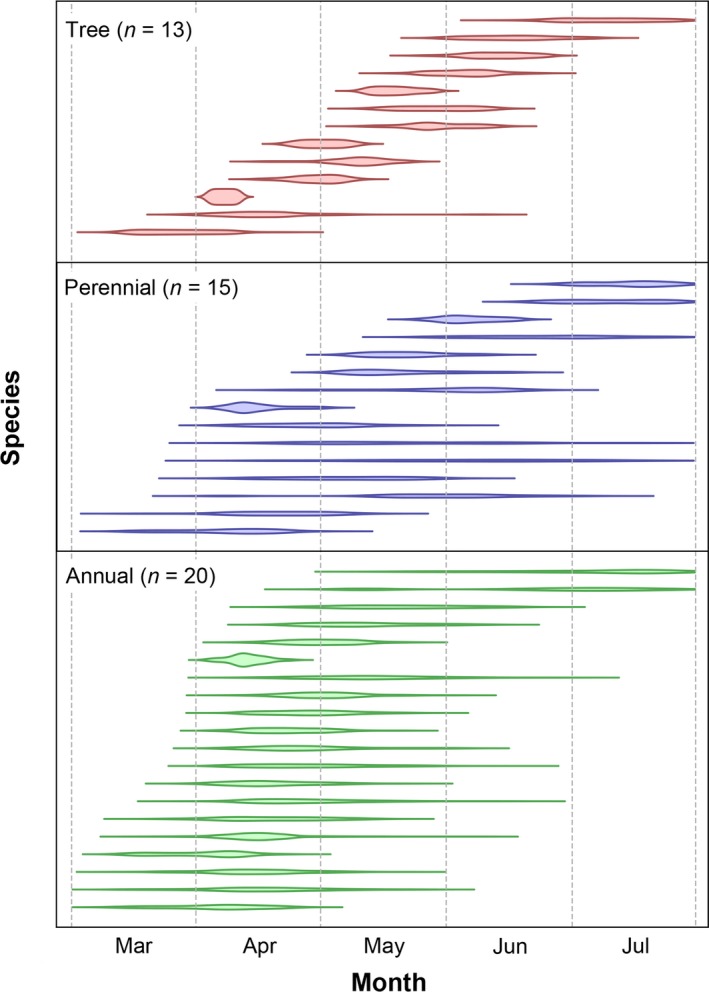
Flowering phenology of insect‐pollinated species. Each line shows the total flowering duration for each species. The tree species (in order of earlier to later flowering): *Eurya japonica* Thunb., *Rubus hirsutus*,* Prunus serrulata*,* Viburnum japonicum* Spreng., *Castanopsis sieboldii*,* Euonymus alatus* (Thunb.) Siebold, *Toxicodendron succedaneum* (L.) Kuntze, *Premna microphylla* Turcz., *Rosa multiflora* Thunb., *Ligustrum japonicum* Thunb., *Cornus macrophylla* Wall., *Trachelospermum asiaticum* Nakai, *Albizia julibrissin*; perennial herbs: *Semiaquilegia adoxoides* Makino, *Lamium album*,* Ranunculus silerifolius* H.Lév. var. *glaber* (H.Boissieu) Tamura, *Cirsium japonicum*,* Trifolium repens*,* Oxalis corniculata* L., *Ranunculus japonicus* Thunb., *Glechoma grandis* (A.Gray) Kprian., *Houttuynia cordata* Thunb., *Clinopodium gracile* (Benth.) Kuntze, *Sisyrinchium rosulatum* E.P.Bicknell, *Erigeron philadelphicus* L., *Sedum bulbiferum*,* Cryptotaenia japonica* Hassk., *Cayratia japonica* Gagnep.; annuals: *Lamium purpureum* L., *Veronica persica* Poir., *Stellaria neglecta* (Lej.) Weihe, *Lamium amplexicaule* L., *Cerastium glomeratum* Thuill., *Stellaria media* (L.) Vill., *Ranunculus muricatus* L., *Corydalis incisa*,* Veronica arvensis* L., *Trigonotis peduncularis* Benth. ex S.Moore & Baker, *Vicia sativa* L. subsp. *nigra* (L.) Ehrh., *Vicia hirsuta* (L.) Gray, *Vicia tetrasperma* (L.) Schreb., *Stellaria aquatica* Scop., *Veronica hederifolia*,* Youngia japonica* (L.) DC., *Geranium carolinianum* L., *Trifolium dubium* Sibth., *Torilis japonica*,* Erigeron annuus* (L.) Pers.

We observed the flowering phenology of 48 insect‐pollinated species (13 species of trees, 15 perennial herbs, and 20 annual herbs; Fig. 2) during the survey period that had five or more individuals in total for both years. Among these species, 43 (11 species of trees, 12 perennial herbs, and 20 annuals) were monitored in both 2016 and 2017. Total flowering length of species (TFL) varied from 9 days in *Prunus serrulata* Lindl. to 48 days in *Albizia julibrissin* Durazz. and 79 days in *Rubus hirsutus* Thunb. in tree species (note that *R. hirsutus* is a small shrub similar to perennial herbs; Appendix S3). In perennial species, TFL ranged from 27 days in *Sedum bulbiferum* Makino to 113 days in *Trifolium repens*; the TFL in annual species ranged from 22 days in *Veronica hederifolia* L. to 89 days in *Torilis japonica* DC. (Fig. 2). The TFL tended to be shorter in trees than in perennial and annual herbs (see Fig. 2; the results of statistical tests are described later). The variance of flowering length of individuals (VFL) was also smaller in trees than in perennial herbs and annual herbs, but mean flowering length of individuals (MFL) was similar among trees, perennial herbs, and annuals. For all life forms, skewness was close to zero and kurtosis above two. Trees tended to have higher *Iδ* values than for perennial herbs or annuals and smaller variance of onset day than for perennial herbs.

Tall trees and shrubs are pollinated by many generalist insect pollinators including hymenopterans, dipterans, lepidopterans, and coleopterans (Appendix S1, including observations by Kuwata, 2013 and ourselves). Three perennial herbs, *Cirsium japonicum* (Asteraceae), *Lamium album* L. (Lamiaceae), and *Trifolium repens* (Fabaceae), were visited by bees (*Bombus ardens*,* Xylocopa appendiculata*, and *Apis mellifera*), while other perennial herbs and annuals were visited mostly by dipterans.

For rarefaction–extrapolation curves used to adjust the number of individuals in statistical tests, the logistic model fitted best for TFL, skewness, and kurtosis, while the logarithmic model fitted best for MFL, VFL, and variance of onset date (Appendices S5, S6). The quadratic model gave the best fit for the aggregation index (Morisita, 1959). In the rarefaction–extrapolation curves, TFL, skewness, and kurtosis increased from 0 to ca. 10 individuals and then leveled off, whereas MFL, VFL, and variance of onset date were almost constant regardless of the number of observed individuals (Appendix S6). The *Iδ* values (Morisita, 1959) increased with the number of observed individuals (Appendix S6).

### Testing for phylogenetic signal

Blomberg's *K* was small and not significantly different from zero for any of the phenological variables (Appendix S7). This result is consistent with the fact that our data for 48 species, 36 genera, and 24 families are not biased toward specific clades. Therefore, we used standard statistical tests assuming random sampling.

### Statistical analysis 1: tests of variables for each species

For the raw data, we first examined the effects of year and the number of observed individuals using a GLMM (or LMM for skewness), in which genus was included as a random factor. The effect of year was not significant for TFL, MFL, VFL, kurtosis, or *Iδ* (Appendix S8). For variance of onset date and skewness, the effect of year was significant (Appendices S8, S9). The effect of the number of observed individuals was significant for TFL, MFL, and *Iδ*; TFL, MFL, and *Iδ* increased when more individuals were observed (Appendix S8).

Because there was no significant effect of year on TFL, MFL, VFL, kurtosis, or *Iδ*, we tested differences in those variables between trees and herbs by pooling the data for 2 years and used a GLMM in which genus was included as a random factor. TFL, MFL, and VFL were significantly smaller in trees, and *Iδ* was significantly larger in trees (Table 2). Kurtosis did not differ between trees and herbs. Using the data for 2 years, we also tested differences in TFL, MFL, VFL, kurtosis, and *Iδ* between annuals and perennial herbs. *Iδ* tended to be larger in annuals, but deviations were marginal (*p* = 0.0509; Table 2). TFL, MFL, VFL, and kurtosis did not differ between annuals and perennial herbs.

**Table 2:** GLMMs examining the effects of life forms on phenological variables. * *P* < 0.05, *** *P* < 0.005.

Phenological variables	Explanatory variables	Intercept	Slope	*P*	
TFL	Tree ‐ Herb	4.07	−0.52	0.00	***
	Perennial ‐ Annual	4.12	−0.06	0.64	
MFL	Tree ‐ Herb	3.18	−0.31	0.03	*
	Perennial ‐ Annual	3.30	−0.20	0.14	
VFL	Tree ‐ Herb	5.31	−1.39	0.00	***
	Perennial ‐ Annual	5.52	−0.29	0.33	
Kurtosis	Tree ‐ Herb	0.95	−0.01	0.95	
	Perennial ‐ Annual	0.94	0.01	0.92	
*Iδ*	Tree ‐ Herb	1.35	0.41	0.02	*
	Perennial ‐ Annual	1.18	0.32	0.05	

**Figure 3:**
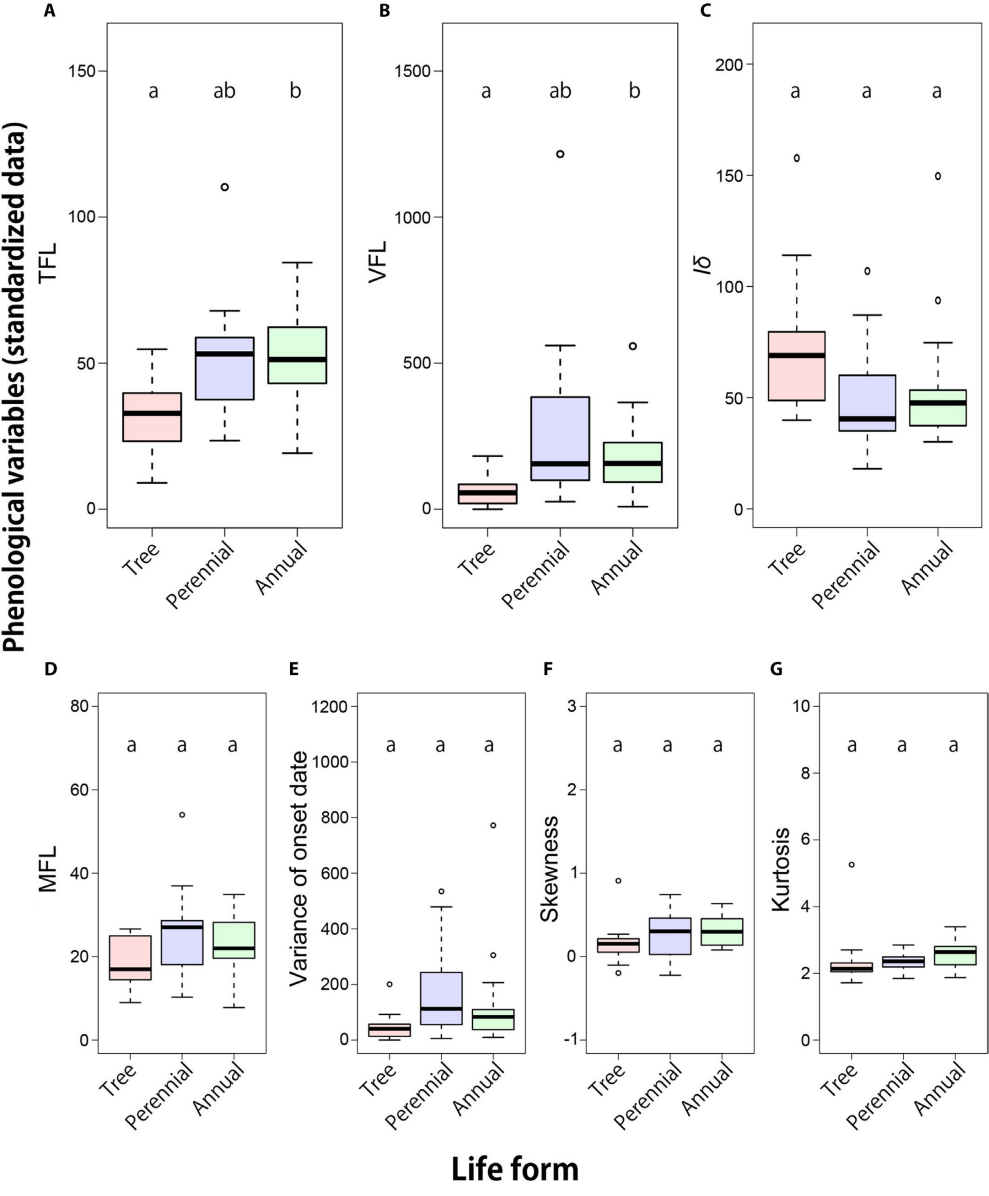
Phenological variables of tree, perennial, and annual species for 12 individuals. (A) TFL: total flowering length, (B) VFL: variance of flowering length, (C) *Iδ*, (D) MFL: mean flowering length, (E) variance of onset dates, (F) skewness, and (G) kurtosis of tree (red box; *n* = 13), perennial herbs (blue box; *n* = 15), and annual herbs (green box; *n* = 20) are shown. The black line inside the box shows the median, the box shows the first quartile to the third quartile, the upper and lower lines show the maximum and minimum values in the range of 1.5 times the length of the box, and the white circles show the outliers. The letters above the boxes indicate their significance; different letters indicate a significant difference. Similar trends were observed in the case of 5, 7, 18, and 22 individuals.

For the data standardized for 5, 7, 12, 18, or 22 individuals, TFL was significantly shorter in trees than in annuals, whereas there was no significant difference between perennial herbs and annuals (Table 3, Fig. 3; Appendices S10, S11). On the other hand, there were no significant differences in MFL among life forms. VFL was significantly smaller in trees than in annuals, whereas there was no significant difference between perennial herbs and annuals. The variance of the onset dates was significantly smaller in trees than in perennial herbs when the raw data were tested but was not significantly different among life forms when standardized values were tested. There were no significant differences in skewness and kurtosis among life forms. *Iδ* was significantly higher in trees than in perennial herbs and annual herbs in both tests using the raw data and the data standardized for 18 or 22 individuals. For the data standardized for 5, 7, and 12 individuals, *Iδ* was not significantly higher in trees than in perennial herbs or annual herbs but deviations in the data standardized for 12 individuals were marginal (*p* = 0.053 for tree vs. perennial comparison, and *p* = 0.053 for tree vs. annual comparison; Table 3).

**Table 3:** Tests among life forms for each phenological variable for 12 individuals, and the raw data. Student's *t* test, Wilcoxon rank sum test, and Fligner‐Policello test were used depending on normality and variance of groups. *P*‐values were adjusted using the Holm method. * *P* < 0.05, *** *P* < 0.005. Similar trends were observed in the case of 5, 7, 18, and 22 individuals (Appendix S13).

Data set		Raw data			*n* = 12		
Phenological variables	Pairs	Statistic	*P*		Statistic	*P*	
TFL	Tree–Perennial	−2.765	0.021	*	65.00	0.285	
	Perennial–Annual	−0.697	0.491		113.00	0.285	
	Annual–Tree	4.470	0.000	***	3.06	0.013	*
MFL	Tree–Perennial	−2.215	0.107		−1.57	0.258	
	Perennial–Annual	0.864	0.394		0.07	0.944	
	Annual–Tree	1.984	0.112		2.19	0.109	
VFL	Tree–Perennial	−4.154	0.000	***	−1.89	0.117	
	Perennial–Annual	0.497	0.497		−0.71	0.479	
	Annual–Tree	4.281	0.000	***	199.00	0.030	*
Variance of onset date	Tree–Perennial	−3.992	0.000	*	−1.73	0.250	
	Perennial–Annual	1.861	0.126		0.44	0.661	
	Annual–Tree	1.829	0.126		1.18	0.479	
Skewness	Tree–Perennial	101.000	0.883		85.00	1.000	
	Perennial–Annual	−1.26	0.437		−0.46	1.000	
	Annual–Tree	176.000	0.277		170.00	0.442	
Kurtosis	Tree–Perennial	−0.690	0.490		−1.27	0.283	
	Perennial–Annual	−1.795	0.145		−1.51	0.283	
	Annual–Tree	187.000	0.108		2.06	0.119	
*Iδ*	Tree–Perennial	5.063	0.000	***	147.00	0.053	
	Perennial–Annual	−1.07	0.286		135.00	0.633	
	Annual–Tree	−3.908	0.000	***	66.00	0.053	

### Statistical analysis 2: tests of variables for interspecies comparison

**Figure 4:**
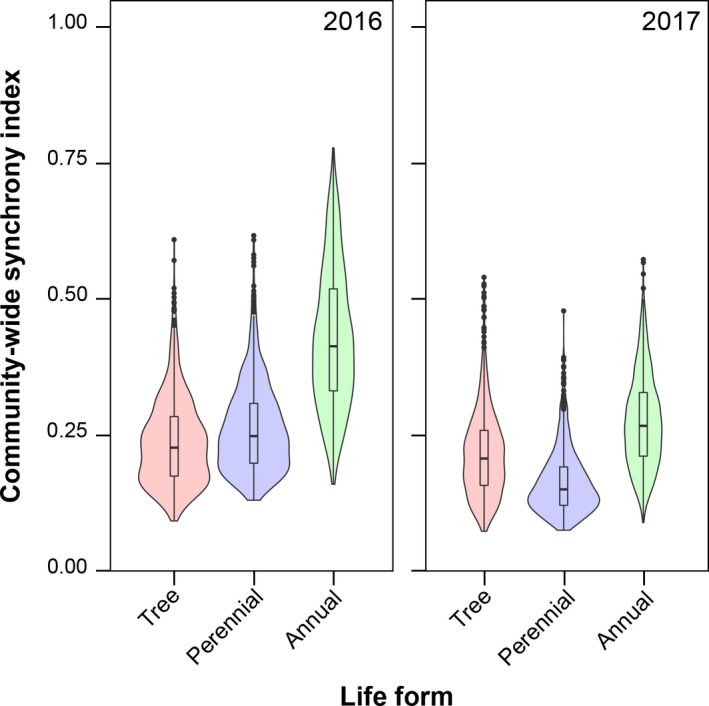
Distributions of the community‐wide synchrony index in trees (red), perennial herbs (blue), and annual herbs (green) in 2016 (left), and in 2017 (right). The black line inside the box shows the median, the box shows the first quartile to the third quartile, the upper and lower lines show the maximum and minimum values in the range of 1.5 times the length of the box, and the black circles show the outliers.

The onset dates varied widely from March to July in both trees and herbs, although relatively more tree species tend to flower in May, more perennial herbs flower in April, and more annuals flower from March to April (Appendix S12). The skewness of the distribution of onset date was positive and significant for annuals in 2016 (Appendices S12, S13), and relatively large and positive skewness values were also found for annuals in 2017 and perennial herbs in 2016. However, bootstrapped skewness distributions largely overlapped, showing that the skewness was not significantly different among life forms (Table 4). The community‐wide synchrony index values were above zero and medians below 0.5 for all life forms; these values were larger for annuals than for perennial herbs and trees in both 2016 and 2017, but the difference was not significant (Table 5, Fig. 4).

**Table 4:** Differences in skewness among life forms.

Form	2016		2017	
	2.50%	97.50%	2.50%	97.50%
Tree	−0.69	0.85	−1.02	0.52
Perennial	−0.61	1.01	−0.61	1.02
Annual	0.5	3.1	−0.12	2.38

**Table 5:** Differences in the community‐wide synchrony index among life forms.

Form	2.50%	97.50%
Tree	0.12	0.426
Perennial	0.15	0.456
Annual	0.219	0.696

## DISCUSSION

### Differences in flowering patterns among trees, perennial herbs, and annuals

This study revealed two significant differences in characteristics of flowering phenology between trees and perennial or annual herbs in a temperate, evergreen–broad‐leaved forest. First, trees have shorter TFL (total flowering length of species) than annual and perennial herbs. However, MFL (mean flowering length of individuals) did not differ significantly among life forms. Second, synchronization of flowering was greater among individual trees than among perennial herbs or annual herbs (larger Morisita's *Iδ* and smaller variance of flowering length of individuals [VFL]). Those results showed that differences of TFL among life forms were derived from differences in VFL and intraspecific synchrony rather than differences in MFL.

While our study in a temperate, evergreen–broad‐leaved forest demonstrated that VFL are smaller and intraspecific synchrony is greater for trees than herbs, another study (Kato et al., 1993) reported that trees flowered for shorter periods than herbs in cool‐temperate deciduous forests, subalpine coniferous forests, and alpine meadows in Japan. However, Kato et al. (1993) only described TFL, and it is uncertain whether the difference of TFL stems from the difference in VFL, intraspecific synchrony, and/or MFL. In a tropical dry forest, Cortés‐Flores et al. (2017) compared TFL between trees and herbs and showed that flowering period was greater for trees than herbs. Descriptions of MFL, VFL, and intraspecific synchrony would be helpful for interpreting this difference from our study. In their seminal review on phenological patterns of terrestrial plants, Rathcke and Lacey (1985) suggested that the phenological pattern is quantitatively described at the levels of individuals, species, and communities by such variables as time of occurrence (onset, mean, mode), duration (range), synchrony (variance), and skewness. Despite this reasonable suggestion, these variables have not been described quantitatively at the levels of individuals, species, and communities until the present study. Here, we established a method to record flowering events at the level of individuals that allows the variables to be calculated at the species and community levels. Below, we compare our observations using the variables with predictions for individual flowering behavior, although further studies using this method are needed to confirm the generality of our findings.

The three hypotheses to explain the differences in flowering phenology among species (Rathcke and Lacey, 1985)—the pollinator attraction hypothesis (Janzen, 1967; Heinrich et al., 1977; Yumoto, 1987, 1988; Cortés‐Flores et al., 2017), the pollination insurance hypothesis (Pojar, 1974), and the resource availability hypothesis (Frankie et al., 1974)—lead to different predictions regarding the relationship between flowering duration and life form (Table 1). We thus next consider which hypothesis better fits the results of our study.

First, the pollinator attraction hypothesis is unlikely to be supported by our observations, given that there was no significant difference in MFL among life forms (Table 6), although intraspecific synchrony was marginally higher in trees as predicted. According to the pollinator attraction hypothesis (Table 1, top), tree individuals are predicted to flower for a shorter duration with greater synchrony to attract more generalist pollinators (Janzen, 1967; Yumoto, 1987, 1988; Cortés‐Flores et al., 2017). This prediction is also derived from an optimization model for the evolution of flowering duration developed by Schoen and Ashman (1995), who suggested that optimal flower longevity is determined by the trade‐off between increasing pollination success by flowering longer and the increasing cost of maintaining flowers. Under this trade‐off, shorter flower longevity will be favored if the return on pollination success is larger but decelerating and the maintenance cost is high. Using the same framework of the optimization model, we predicted that MFL would be shorter in trees than in herbs because trees attract many generalist pollinators (as is the case in temperate forest trees; Yumoto, 1987, 1988) by flowering more abundantly than herbs. Our observations did not agree with this prediction.

**Table 6:** Results of each phenological variable for each life form.

Phenological variables	Trees	Perennial herbs	Annuals	Support for hypotheses
TFL	Shorter	Intermediate (ns)	Longer	The three hypotheses are not relevant because they make predictions for individuals.
MFL	(ns)	(ns)	(ns)	None of the hypotheses were supported.
VFL	Smaller	Intermediate (ns)	Larger	Resource availability hypothesis was supported only for annuals.
Synchronicity (*Iδ*)	Higher (ns)	Lower (ns)	Lower (ns)	Pollinator attraction hypothesis was supported only marginally.

Second, the pollination insurance hypothesis does not appear to be supported by our observations for MFL (Table 6). According to the pollination insurance hypothesis (Table 1 middle), selfing annuals are expected to flower for shorter durations because fertilization is ensured by selfing. While annual species do not necessarily self‐fertilize (Aarssen, 2000), most annual weeds are able to set seeds by autogamy (Baker, 1974). In colonizers such as annual weeds, an ability to self‐fertilize ovules is more advantageous than outcrossing because colonizers often lack compatible mates and fewer pollinators are present (Pannell, 2015). In this study, although most of annuals we observed were colonizing weeds, MFL did not differ between annuals and other life forms.

Third, our observations provided mixed support for the resource availability hypothesis (Table 6). According to the resource availability hypothesis (Table 1, below), (1) in unpredictable habitats, annuals should flower earlier and longer, with larger variance (Harper and White, 1974; Grime, 1977; Sakai and Harada, 1993; Masuda and Yahara, 1994; Klimesšová et al., 2016), and (2) in predictable habitats, larger plants flower longer for all life forms (Samson and Werk, 1986; Bazzaz et al., 1987; Fabbro and Korner, 2004), and consequently trees should show larger MFL and VFL than perennial and annual herbs. Among these possibilities, the prediction for plants in unpredictable habitats was supported by our observation that VFL was larger in annuals. The prediction for reduced MFL and greater VFL for plants of predictable habitats, however, did not agree with our observations that MFL did not differ among life forms and VFL was smaller in trees than herbs.

We suggest that the smaller VFL of trees relative to perennial and annual herbs may be a mechanism to increase outcrossing by promoting between‐tree movement of pollinators. Because individual trees have many flowers, there is a higher risk of geitonogamy if pollinators stay longer in one tree. According to the theoretical and empirical study of Ohashi and Yahara (2002), a higher density of simultaneously flowering plants promotes between‐individual movement of pollinators because the energetic costs of between‐individual movement relative to within‐individual movement is lower under a higher density of flowering individuals. Therefore, natural selection would favor more accurate detection of cues that enable individual trees to synchronize flowering among conspecific individuals within a population to promote between‐individual movement of pollinators. This hypothesis, here designated as a modified pollinator attraction hypothesis, explains our finding that VFL is smaller in trees than in herbs. Yumoto (1987) also showed that flowering canopy tree species showed higher intraspecific synchrony (larger Morisita's *Iδ*) than did flowering understory‐tree species. This finding is consistent with our view because canopy tree species have more flowers and a higher risk of geitonogamy than do understory tree species.

### Community‐wide flowering patterns

There were no significant differences in the variance of onset date and community‐wide interspecific synchrony between trees, perennial herbs, and annuals. Interspecific synchrony, however, was greater than zero in all life forms (Fig. 4), indicating that flowering events are weakly synchronized. In another comparison of flowering patterns between trees and herbs (Kato et al., 1993), flowering durations in both trees and herbs largely overlapped among successively flowering species, but community‐wide interspecific synchrony was not determined.

Community‐wide interspecific synchrony has been reported also for tropical forest where rainfall varies seasonally: more trees flowered during late dry and early wet seasons, whereas more herbs flowered during late wet season (Batalha and Martins, 2004, in tropical wet forest; Joshi and Janarthanam, 2004, in plateaus, moist deciduous forest, semi‐evergreen forest, evergreen forest, and mangroves; Monasterio and Sarmiento, 1976, in tropical savanna and the semi‐deciduous forest). On the other hand, the weak flowering synchrony in all life forms of temperate forests may be explained by the existence of winter, a period not suitable for growth and flowering (see Doi et al., 2008; Forrest, 2015; Inouye, 2008).

## CONCLUSIONS

In conclusion, the differences in flowering phenology variables (TFL, VFL and intraspecies synchrony) among trees, perennial herbs, and annuals are likely to be explained by the modified pollinator attraction hypothesis for trees, and the resource availability hypothesis in unpredictable habitats for annuals. On the other hand, weak but positive interspecific synchrony supports that flowering times tend to converge rather than diverge between species. These conclusions are derived from quantitative observations of the flowering phenology of individual plants, enabling comparisons of TFL, MFL, VFL, and interspecies synchrony among trees, perennial herbs, and annual herbs. Further quantitative studies using this protocol are needed to determine whether similar patterns are observed in plant communities under different climatic conditions. We showed that TFL and VFL varied among life forms but MFL did not. This result suggests that phenological responses to environmental changes, such as earlier emergence of pollinators due to global warming (Elzinga et al., 2007), would occur through changes in TFL and VFL rather than changes in MFL, and those responses would differ among life forms. In recent years, possibly reflecting environmental fluctuations due to climate change, phenological fluctuations associated with pollinator–plant interactions (Parmesan, 2006; Hegland et al., 2009) and plant–plant interactions have been reported (Sparks et al., 2000; Dunne et al., 2003; Miller‐Rushing et al., 2006; Forrest et al., 2010; Cara‐Donna et al., 2014; Heberling et al., 2019). To deepen our understanding of those phenological responses to climate change, we need additional detailed studies of phenology to determine TFL, MFL, and VFL and interspecific synchrony for different life forms.

## AUTHOR CONTRIBUTIONS

T.Y. conceived the research idea; A.N. designed the research and collected data; A.N. and T.Y. analyzed data; T.Y. supervised A.N. in writing the manuscript. All authors contributed critically to the drafts and gave final approval for publication.

## Supporting information


**APPENDIX S1.** Details of pollinators observed in trees.

**APPENDIX S2.** Plant species list.

**APPENDIX S3.** Annual fluctuations in temperature and precipitation.

**APPENDIX S4.** Information on DNA sequences used to construct phylogenetic tree.

**APPENDIX S5.** Rarefaction–extrapolation curves for seven phenological variables.

**APPENDIX S6.** Model selection using Bayesian information criterion (BIC).

**APPENDIX S7.** Tests of phylogenetic signals.

**APPENDIX S8.** GLMMs examining the effects of year and the number of observed individuals on phenological variables.

**APPENDIX S9.** LMM examining the effects of year and the number of observed individuals on skewness.

**APPENDIX S10.** Tests among life forms for each phenological variable.

**APPENDIX S11.** The results of comparing each phenological variable for the species among life forms in the case of raw data.

**APPENDIX S12.** Distributions of onset date in trees, perennial herbs, and annual herbs.

**APPENDIX S13.** Differences in the distributions of onset date from normal.
