# Supplementary material for: Quantitative comparison of flowering phenology traits among trees, perennial herbs, and annuals in a temperate plant community
Source: Am J Bot. 2019 Nov 14;106(12):1545–57. doi: 10.1002/ajb2.1387 (PMC6973048; doi:10.1002/ajb2.1387)
Supplement: Supplementary file 1 — APPENDIX S1. Details of pollinators observed in trees. [file AJB2-106-1545-s001.docx]

| **Family** | **Observed species** | **Life form** | **Method** | **Hymenoptera** | **Diptera** | **Lepidoptera** | **Coleoptera** | **Total** |
| --- | --- | --- | --- | --- | --- | --- | --- | --- |
| Adoxaceae | *Viburnum japonicum* Spreng. | Shrub | Camera | 1 | 33 | 0 | 181 | 220 |
| Apocynaceae | *Trachelospermum asiaticum* Nakai | Woody vine | Camera | 0 | 1 | 6 | 0 | 7 |
| Celastraceae | *Euonymus alatus* (Thunb.) Siebold | Shrub | Camera | 2 | 5 | 0 | 0 | 8 |
|  |  |  | Direct | 22 | 30 | 0 | 14 | 78 |
| Rosaceae | *Rosa multiflora* Thunb. | Shrub | Camera | 1 | 4 | 0 | 5 | 13 |
|  |  |  | Direct | 63 | 8 | 0 | 1 | 72 |
| Rosaceae | *Rubus hirsutus* Thunb. | Shrub | Camera | 6 | 7 | 7 | 4 | 25 |
| Ternstroemiaceae | *Eurya japonica* Thunb. | Shrub | Camera | 0 | 18 | 7 | 4 | 29 |
| Asteraceae | *Cirsium japonicum* DC. | Perenial herb | Direct | 36 | 10 | 15 | 18 | 85 |
| Anacardiaceae | *Toxicodendron succedaneum* (L.) Kuntze | Tall tree | Camera | 0 | 14 | 7 | 11 | 32 |
|  |  |  | Direct | 11 | 22 | 1 | 15 | 49 |
| Cornaceae | *Cornus macrophylla* Wall. | Tall tree | Camera | 0 | 1 | 1 | 16 | 24 |
|  |  |  | Direct | 21 | 25 | 2 | 13 | 73 |
| Fabaceae | *Albizia julibrissin* Durazz. | Tall tree | Camera | 1 | 0 | 4 | 5 | 11 |
| Fagaceae | *Castanopsis sieboldii* (Makino) Hatus. | Tall tree | Camera | 2 | 16 | 0 | 68 | 95 |
| Oleaceae | *Ligustrum japonicum* Thunb. | Tall tree | Camera | 9 | 5 | 7 | 5 | 35 |
|  |  |  | Direct | 47 | 11 | 10 | 0 | 71 |
| Rosaceae | *Prunus serrulata* Lindl. | Tall tree | Camera | 0 | 11 | 0 | 7 | 20 |

**Appendix S1. Details on pollinators observed in trees.** Insect flower visitors were monitored by taking a photograph every10 min using programmable digital cameras for trees (Camera; Kuwata 2013) or direct visual observation (Direct).
