# Supplementary material for: Quantitative comparison of flowering phenology traits among trees, perennial herbs, and annuals in a temperate plant community
Source: Am J Bot. 2019 Nov 14;106(12):1545–57. doi: 10.1002/ajb2.1387 (PMC6973048; doi:10.1002/ajb2.1387)
Supplement: Supplementary file 2 — APPENDIX S2. Plant species list. [file AJB2-106-1545-s002.docx]

**Appendix S2. Details on plant species monitored**.

| **Family** | **Species** | **Life form** | **Weediness** | **Reproductive system** |
| --- | --- | --- | --- | --- |
| Adoxaceae | *Viburnum japonicum* Spreng. | Tree | 0 | Outcrossing^3^ |
| Anacardiaceae | *Toxicodendron succedaneum* (L.) Kuntze | Tree | 0 | Outcrossing^3^ |
| Apocynaceae | *Trachelospermum asiaticum* Nakai | Tree | 0 | Outcrossing^3^ |
| Celastraceae | *Euonymus alatus* (Thunb.) Siebold | Tree | 0 | Insect^3^ |
| Cornaceae | *Cornus macrophylla* Wall. | Tree | 0 | Insect^3^ |
| Fabaceae | *Albizia julibrissin* Durazz. | Tree | 0 | Insect^3^ |
| Fagaceae | *Castanopsis sieboldii* (Makino) Hatus. | Tree | 0 | Insect^3^ |
| Lamiaceae | *Premna microphylla* Turcz. | Tree | 0 | Insect^3^ |
| Oleaceae | *Ligustrum japonicum* Thunb. | Tree | 0 | Insect^3^ |
| Pentaphylacaceae | *Eurya japonica* Thunb. | Tree | 0 | Insect^3^ |
| Rosaceae | *Prunus serrulata* Lindl. | Tree | 0 | Insect^3^ |
| Rosaceae | *Rosa multiflora* Thunb. | Tree | 0 | Insect^3^ |
| Rosaceae | *Rubus hirsutus* Thunb. | Tree* | 0 | Insect^3^ |
| Apiaceae | *Cryptotaenia japonica* Hassk. | Perennial | 0 | Insect^4^ |
| Asteraceae | *Cirsium japonicum* DC. | Perennial | Roadside^1^ | Insect^4^ |
| Asteraceae | *Erigeron philadelphicus* L. | Perennial | Arable^2^ | Insect^4^ |
| Crassulaceae | *Sedum bulbiferum* Makino | Perennial | Roadside^1^ | Vegetable reproduction |
| Fabaceae | *Trifolium repens* L. | Perennial | Arable^2^ | Insect^4^ |
| Iridaceae | *Sisyrinchium rosulatum* E.P.Bicknell | Perennial | Arable^2^ | Insect^4^ |
| Lamiaceae | *Clinopodium gracile* (Benth.) Kuntze | Perennial | Roadside^1^ | Insect^4^ |
| Lamiaceae | *Glechoma grandis* (A.Gray) Kuprian | Perennial | Roadside^1^ | Insect^4^ |
| Lamiaceae | *Lamium album* L. | Perennial | 0 | Insect^4^ |
| Oxalidaceae | *Oxalis corniculata* L. | Perennial | Arable^2^ | Insect^4^ |
| Ranunculaceae | *Ranunculus japonicus* Thunb. | Perennial | 0 | Insect^4^ |
| Ranunculaceae | *Ranunculus silerifolius* H.Lév. var. *glaber* (H.Boissieu) Tamura | Perennial | 0 | Insect^4^ |
| Ranunculaceae | *Semiaquilegia adoxoides* Makino | Perennial | 0 | Selfing^4^ |
| Saururaceae | *Houttuynia cordata* Thunb. | Perennial | Roadside^1^ | Agamospermy^4^ |
| Vitaceae | *Cayratia japonica* Gagnep. | Perennial | Roadside^1^ | Insect^4^ |
| Apiaceae | *Torilis japonica* DC. | Annual | Arable^2^ | Insect^4^ |
| Asteraceae | *Erigeron annuus* (L.) Pers. | Annual | Arable^2^ | Agamospermy^4^ |
| Asteraceae | *Youngia japonica* (L.) DC. | Annual | Arable^2^ | Insect^4^ |
| Boraginaceae | *Trigonotis peduncularis* Benth. ex S.Moore & Baker | Annual | Arable^2^ | Selfing^4^ |
| Caryophyllaceae | *Cerastium glomeratum* Thuill. | Annual | Arable^2^ | Selfing^4^ |
| Caryophyllaceae | *Stellaria aquatica* Scop. | Annual | Arable^2^ | Insect/selfing^4^ |
| Caryophyllaceae | *Stellaria media* (L.) Vill. | Annual | Arable^2^ | Selfing^4^ |
| Caryophyllaceae | *Stellaria neglecta* (Lej.) Weihe | Annual | Roadside^1^ | Insect/selfing^4^ |
| Fabaceae | *Trifolium dubium* Sibth. | Annual | Arable^2^ | Insect^4^ |
| Fabaceae | *Vicia hirsute* (L.) Gray | Annual | Arable^2^ | Selfing^4^ |
| Fabaceae | *Vicia sativa* L. subsp. *nigra* (L.) Ehrh. | Annual | Arable^2^ | Insect^4^ |
| Fabaceae | *Vicia tetrasperma* (L.) Schreb. | Annual | Arable^2^ | Insect^4^ |
| Geraniaceae | *Geranium carolinianum* L. | Annual | Arable^2^ | Selfing^4^ |
| Lamiaceae | *Lamium amplexicaule* L. | Annual | Arable^2^ | Insect^4^ |
| Lamiaceae | *Lamium purpureum* L. | Annual | Arable^2^ | Selfing^4^ |
| Papaveraceae | *Corydalis incisa* Pers. | Annual | 0 | Insect^4^ |
| Plantaginaceae | *Veronica arvensis* L. | Annual | Arable^2^ | Selfing^4^ |
| Plantaginaceae | *Veronica hederifolia* L. | Annual | Arable^2^ | Insect/selfing^5^ |
| Plantaginaceae | *Veronica persica* Poir. | Annual | Arable^2^ | Insect/selfing^5^ |
| Ranunculaceae | *Ranunculus muricatus* L. | Annual | Roadside^1^ | Insect^4^ |

*For the life form of *Rubus hirsutus*, we used the classification of Naruhashi and Terao (1978)^6^, who described it as suffruticose or a small shrub. It is intermediate between a tree and herb in that stem longevity is from 1 to 1.5 years. Also, its flowering duration is the longest among tree species (Fig. 2). Including *R. hirsutus* as a perennial herbs was more advantageous for our conclusion, so to be conservative, we included it as a tree.

**Literature Cited**

1. Asai, M. 2016. The handbook of weed seedlings 2. Bunichi-sogo-shuppan, Tokyo, Japan (in Japanese).

2. Asai, M. 2012. The handbook of weed seedlings. Bunichi-sogo-shuppan, Tokyo, Japan (in Japanese).

3. Kuwata K. 2013. Flowering phenology and pollinators of 44 tree species in a stand translocated under a forest restoration project. M.S. thesis, Kyushu University, Fukuoka, Japan.

4. Yahara, T., A. Nagahama et al. unpublished.

5. Tsuruuchi, T. 1994. Reproductive ecology of *Veronica hederaefolia* L. and *V. persica* Poir. *Weed Research* 39: 85-90 (in Japanese with English abstract).

6. Naruhashi, N. and Terao, K. 1978. Seasonal changes in the proportional distributions of dry matter into various organs of *Rubus hirsutus*. *Journal of Geobotany* 26: 74–80.
