# Supplementary material for: Quantitative comparison of flowering phenology traits among trees, perennial herbs, and annuals in a temperate plant community
Source: Am J Bot. 2019 Nov 14;106(12):1545–57. doi: 10.1002/ajb2.1387 (PMC6973048; doi:10.1002/ajb2.1387)
Supplement: Supplementary file 3 — APPENDIX S3. Annual fluctuations in temperature and precipitation. [file AJB2-106-1545-s003.docx]

(a)

(b)

**Appendix S3. Monthly mean temperature (a) and precipitation (b) in 2016 in the biodiversity reserve of Ito campus, Kyushu University, Fukuoka, Japan.** Monthly average temperature ranged from 6.2°C in January to 27.4°C in August (range: 21.2°C) and monthly precipitation ranged from 75.5 mm in March to 337 mm in June (range: 261.5 mm). We removed the precipitation data in September 2016 because of equipment failure. Data were recorded by Kyushu University (2018).
