# Supplementary material for: Quantitative comparison of flowering phenology traits among trees, perennial herbs, and annuals in a temperate plant community
Source: Am J Bot. 2019 Nov 14;106(12):1545–57. doi: 10.1002/ajb2.1387 (PMC6973048; doi:10.1002/ajb2.1387)
Supplement: Supplementary file 4 — APPENDIX S4. Information on DNA sequences used to construct phylogenetic tree. [file AJB2-106-1545-s004.docx]

**Appendix S4.** DNA sequences used to construct phylogenetic trees. Species in bold were used for all analyses.

| **Family** | **Species** | **Accession** | |
| --- | --- | --- | --- |
|  |  | ***rbcL*** | ***matK*** |
| Adoxaceae | *Sambucus racemosa* L. subsp. *sieboldiana* Blume ex Miq. | AB586169 | HQ714381 |
|  | ***Viburnum japonicum*** Spreng. | HQ591733 | HQ591592 |
|  | *Viburnum awabuki* K.Koch | HQ591704 | JF95680 |
| Anacardiaceae | *Rhus chinensis* Mill. | GQ436548 | KP093558 |
|  | ***Toxicodendron succedaneum*** (L.) Kuntze | AB983117 | KP093670 |
| Apiaceae | *Chamaele decumbens* Makino | D44560 | - |
|  | ***Cryptotaenia japonica*** Hassk. | DQ006050 | HQ593258 |
|  | *Osmorhiza aristata* (Thunb.) Rydb. | D44578 | JF954794 |
|  | ***Torilis japonica*** DC. | D44590 | JN895299 |
| Apocynaceae | ***Trachelospermum asiaticum*** Nakai | GQ436400 | EF456324 |
| Aquifoliaceae | *Ilex integra* Thunb. | FJ394619 | KJ687631 |
|  | *Ilex rotunda* Thunb. | JN407236 | HQ415255 |
| Araceae | *Arisaema ringens* Schott | KT025754 | KT025804 |
| Araliaceae | *Aralia elata* (Miq.) Seem. | KF412439 | KF412425 |
| Asparagaceae | *Liriope muscari* L.H.Bailey | KC704932 | AB029784 |
| Asteraceae | ***Cirsium japonicum*** DC. | GQ436443 | HM989744 |
| Asteraceae | ***Erigeron annuus*** (L.) Pers. | AB851488 | HM989796 |
|  | ***Erigeron philadelphicus*** L. | JX848412 | HQ593287 |
|  | *Gamochaeta pensylvanica* (Willd.) Cabrera | EU384977 | EU385354 |
|  | *Ixeris japonica* Nakai | KX527023 | KX526560 |
|  | *Sonchus oleraceus* L. | KF196024 | KF195980 |
|  | *Taraxacum officinale* F.H.Wigg. | KM361005 | AJ633157 |
|  | ***Youngia japonica*** (L.) DC. | HQ644085 | HM989752 |
| Betulaceae | *Alnus sieboldiana* Matsum. | AB060562 | AB060053 |
| Boraginaceae | ***Trigonotis peduncularis*** Benth. ex S.Moore & Baker | AB744074 | - |
| Brassicaceae | *Capsella bursa-pastoris* (L.) Medik. | D88904 | KF923122 |
|  | *Cardamine scutata* Thunb. | JF941125 | JF953420 |
|  | *Lepidium virginicum* L. | D88906 | HM850737 |
|  | *Rorippa indica* (L.) Hiern | D88907 | AF144355 |
| Campanulaceae | *Triodanis perfoliata* (L.) Nieuwl. | EU713363 | EU713256 |
| Cannabaceae | *Celtis sinensis* Pers. | LC050727 | AF345316 |
| Caprifoliaceae | *Lonicera japonica* Thunb. | HM228498 | HM228455 |
| Caryophyllaceae | *Arenaria serpyllifolia* L. | KM360648 | AY936304 |
|  | *Cerastium fontanum* Baumg. subsp. *vulgare* (Hartm.) Greuter & Burdet | KM360705 | JN893959 |
|  | ***Cerastium glomeratum*** Thuill. | HM849882 | JN895359 |
|  | ***Stellaria aquatica*** Scop. | KM360890 | FJ404855 |
|  | ***Stellaria media*** (L.) Vill. | M62570 | KP642870 |
|  | ***Stellaria neglecta*** (Lej.) Weihe | JN892188 | JN893860 |
|  | *Stellaria alsine* Hoffm. subsp. *undulata* (Thunb.) Vorosch. | KC484153 | HM850778 |
| Celastraceae | *Celastrus orbiculatus* Thunb. | LC006125 | LC006126 |
|  | ***Euonymus alatus*** (Thunb.) Siebold | AB233942 AF530905 | EF135537 |
| Commelinaceae | *Commelina communis* L. | JX903248 | GQ434279 |
|  | *Pollia japonica* Thunb. | KM895510 | FR832815 |
| Cornaceae | ***Cornus macrophylla*** Wall. | AF190433 | DQ340461 |
| Crassulaceae | ***Sedum bulbiferum*** Makino | GQ436423 | AF115652 |
| Euphorbiaceae | *Mallotus japonicus* (L.f.) Müll.Arg. | AB267923 AF530857 | EF582649 |
| Fabaceae | ***Albizia julibrissin*** Durazz. | Z70147 | AY386855 |
|  | *Astragalus sinicus* L. | LN873177 | AY920450 |
|  | *Lotus japonicus* (Regel) K.Larsen | KM372981 | KM372671 |
|  | *Medicago polymorpha* L. | KJ773677 | JX505826 |
|  | *Robinia pseudoacacia* L. | U74220 | HM049518 |
|  | ***Trifolium dubium*** Sibth. | HM850412 | AF522121 |
|  | ***Trifolium repens*** L. | GQ436346 | AF522131 |
|  | ***Vicia hirsuta*** (L.) Gray | KP896752 | AF522157 |
|  | ***Vicia sativa*** L. subsp. ***nigra*** (L.) Ehrh. | AB517630 | JX505840 |
|  | ***Vicia tetrasperma*** (L.) Schreb. | KP896751 | HM026384 |
|  | *Wisteria brachybotrys* Siebold & Zucc | AB729100 | EU424078 |
| Fagaceae | ***Castanopsis sieboldii*** (Makino) Hatus. | AB060564 | AB060055 |
|  | *Lithocarpus edulis* Nakai | AB060569 | AB060060 |
|  | *Quercus acutissima* Carruth. | AB060578 | AB060069 |
|  | *Quercus glauca* Thunb. | AB060571 | AB060062 |
|  | *Quercus serrata* Murray | AB060576 | AB060067 |
|  | *Quercus variabilis* Blume | AB060574 | AB060065 |
| Garryaceae | *Aucuba japonica* Thunb. | AY725858 | AJ429318 |
| Geraniaceae | ***Geranium carolinianum*** L. | JF941753 | EU922172 |
|  | *Geranium thunbergii* Siebold ex Lindl. & Paxton | JF941758 | JF953875 |
| Hydrangeaceae | *Deutzia crenata* Siebold & Zucc. | JF308656 | KP120222 |
| Iridaceae | *Sisyrinchium angustifolium* Mill. | JQ670565 | JQ670487 |
|  | ***Sisyrinchium rosulatum*** E.P.Bicknell | AB744223 | HQ606747 |
| Lamiaceae | *Ajuga decumbens* Thunb. | JQ322527 | AF315299 |
|  | *Callicarpa japonica* Thunb. | JQ618479 | FM163257 |
|  | *Callicarpa mollis* Siebold & Zucc. | HQ384868 | HQ384498 |
|  | ***Clinopodium gracile*** (Benth.) Kuntze | KX527227 | KX526669 |
|  | ***Glechoma grandis*** (A.Gray) Kprian. | AB266226 | HQ593314 |
|  | ***Lamium album*** L. | KM360840 | AJ429332 |
|  | ***Lamium amplexicaule*** L. | AB266225 | JN894206 |
|  | ***Lamium purpureum*** L. | AB266224 | HQ384493 |
|  | ***Premna microphylla*** Turcz. | U28883 | HQ427331 |
|  | *Salvia plebeia* R.Br. | AB295077 | JQ934085 |
| Lardizabalaceae | *Akebia quinata* (Thunb. ex Houtt.) Decne. | GQ436540 | AF542587 |
| Lardizabalaceae | *Stauntonia hexaphylla* Decne. | D85694 | FJ626517 |
| Lauraceae | *Cinnamomum yabunikkei* H.Ohba | HM019460 | KF740405 |
| Liliaceae | *Cardiocrinum cordatum* Makino | AB034918 | AB049523 |
| Menispermaceae | *Cocculus trilobus* DC. | D85696 | DQ478611 |
| Moraceae | *Morus alba* L. | KC584883 | AY257531 |
| Myricaceae | *Morella rubra* Lour. | KF418924 | KF419021 |
| Oleaceae | *Forsythia suspensa* Vahl | GQ436541 | FJ263956 |
|  | ***Ligustrum japonicum*** Thunb. | JF830477 | JF830553 |
| Onagraceae | *Oenothera laciniata* Hill. | KJ773700 | KJ772960 |
| Orchidaceae | *Spiranthes sinensis* (Pers.) Ames | JF972913 | JF972946 |
| Orobanchaceae | *Bellardia viscosa* (L.) Fisch. & C.A.Mey | KM360915 | AY849606 |
| Oxalidaceae | ***Oxalis corniculata*** L. | AB233943_AF530906 | AB233839 |
|  | *Oxalis debilis* Kunth subsp. *corymbosa* (DC.) O.Bolòs & Vigo | KJ773708 | HM851018 |
|  | *Oxalis dillenii* Jacq. | L01938 | KT456915 |
| Papaveraceae | ***Corydalis incisa*** Pers. | KX272421 | KU362910 |
| Phrymaceae | *Mazus miquelii* Makino | HQ384872 | HQ384502 |
|  | *Mazus pumilus* (Burm.f.) Steenis | FJ172728 | HM850959 |
| Pittosporaceae | *Pittosporum tobira* W.T.Aiton | D44582 | HQ619824 |
| Plantaginaceae | *Nuttallanthus canadensis* (L.) D.A.Sutton | KJ773632 | KJ772895 |
|  | *Plantago asiatica* L. | GQ436317 | GQ434075 |
|  | *Plantago virginica* L. | KJ773757 | KJ773014 |
|  | ***Veronica arvensis*** L. | HM850447 | AF052003 |
|  | ***Veronica hederifolia*** L. | KP402621 | JN894703 |
|  | ***Veronica persica*** Poir. | HM850452 | HQ384536 |
| Polygonaceae | *Fallopia japonica* (Houtt.) Ronse Decr. | JF950004 | EU024772 |
|  | *Persicaria longiseta* (Bruijn) Kitag. | FM883631 | EU196943 |
|  | *Persicaria sagittata* (L.) H.Gross | EF653773 | KJ840962 |
|  | *Persicaria thunbergii* (Siebold & Zucc.) H.Gross | HQ435356 | EF653719 |
|  | *Rumex acetosa* L. | KX095189 | KX095187 |
|  | *Rumex japonicus* Houtt. | AB744072 | GQ434138 |
| Primulaceae | *Lysimachia japonica* Thunb. | KJ841403 | JN895201 |
| Ranunculaceae | ***Ranunculus japonicus*** Thunb. | FJ449862 | AY954200 |
|  | ***Ranunculus muricatus*** L. | HM850296 | AY954191 |
|  | *Ranunculus sceleratus* L. | AB517148 | GU257993 |
|  | ***Ranunculus silerifolius*** H.Lév. var. ***glaber*** (H.Boissieu) Tamura | FJ449861 | HQ338367 |
|  | ***Semiaquilegia adoxoides*** Makino | EF437147 | EF437137 |
| Rosaceae | ***Prunus serrulata*** Lindl. | AB729085 | KP760073 |
|  | *Potentilla anemonifolia* Lehm. | GQ436578 | GQ434187 |
|  | *Potentilla indica* (Andrews) Th.Wolf | KX527251 | KT808472 |
|  | *Rhaphiolepis indica* (L.) Lindl. var. *umbellata* (Thunb. ex Murray) H.Ohashi | AB936040 | AB936041 |
|  | ***Rosa multiflora*** Thunb. | KP402729 | FJ472524 |
|  | *Rosa multiflora* Thunb. var. *adenochaeta* (Koidz.) Ohwi ex H.Ohba | - | AB039305 |
|  | *Rosa sambucina* Koidz. | KP095034 | AB039306 |
|  | ***Rubus hirsutus*** Thunb. | GU363792 | JN566120 |
|  | *Rubus parvifolius* L. | GU363802 | AB073699 |
| Rubiaceae | *Galium kikumugura* Ohwi | JX848534 | HQ593306 |
|  | *Galium spurium* L. | KM980627 | KJ204484 |
|  | *Paederia foetida* L. | KC305913 | AY538409 |
| Salicaceae | *Salix triandra* L. | FJ788587 | EU790687 |
| Saururaceae | ***Houttuynia cordata*** Thunb. | AB205610 | AF543737 |
|  | *Saururus chinensis* Hort. ex Loudon | AF332101 | GQ434225 |
| Schisandraceae | *Kadsura japonica* (L.) Dunal | KP689922 | AF542565 |
| Smilacaceae | *Smilax china* L. | D28333 | AB040204 |
| Staphyleaceae | *Euscaphis japonica* (Thunb.) Kanitz | DQ307099 | DQ663628 |
| Styracaceae | *Styrax japonica* Siebold & Zucc. | Z80189 | - |
| Symplocaceae | *Symplocos kuroki* Nagam. | AB729084 | AB925051 |
| Ternstroemiaceae | ***Eurya japonica*** Thunb. | AF380039 | AF380081 |
| Theaceae | *Camellia japonica* L. | AF380035 | KU054403 |
| Urticaceae | *Boehmeria nivea* (L.) Gaudich. | AB125345 | KP093304 |
| Verbenaceae | *Verbena brasiliensis* Vell. | HQ644080 | GQ434146 |
| Violaceae | *Viola japonica* Langsd. ex Ging. | JQ950626 | DQ842592 |
|  | *Viola verecunda* A.Gray | JQ950629 | DQ842580 |
| Vitaceae | *Ampelopsis glandulosa* (Wall.) Momiy. var. *heterophylla* (Thunb.) Momiy. | KT006333 | KX526800 |
|  | ***Cayratia japonica*** Gagnep. | AB851492 | KX526802 |
