# Supplementary material for: Quantitative comparison of flowering phenology traits among trees, perennial herbs, and annuals in a temperate plant community
Source: Am J Bot. 2019 Nov 14;106(12):1545–57. doi: 10.1002/ajb2.1387 (PMC6973048; doi:10.1002/ajb2.1387)
Supplement: Supplementary file 5 — APPENDIX S5. Rarefaction–extrapolation curves for seven phenological variables. [file AJB2-106-1545-s005.docx]

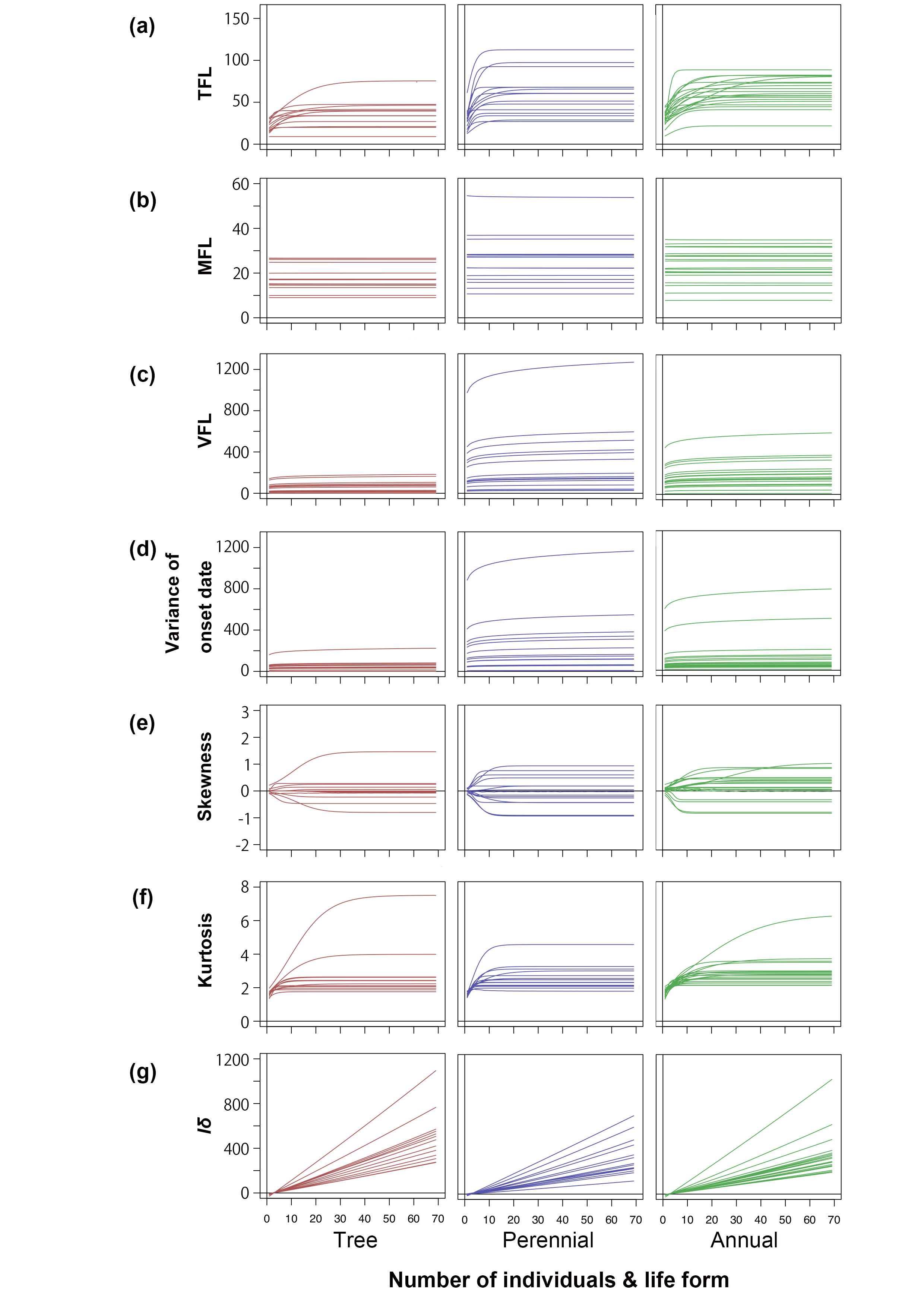


**Appendix S5.** **Rarefaction–extrapolation curves for seven phenological variables for 13 species of trees, 15 perennial herbs, and 20 annuals.** (a) TFL: total flowering length, (b) MFL: mean flowering length, (c) VFL: variance of flowering length, (d) variance of onset dates, (e) skewness, (f) kurtosis (the deviation from normal distribution and describes the weight of the distribution tail), (g) Morisita aggregation index (*Iδ*; larger values represent higher synchrony among individuals)*.*
