# Supplementary material for: Quantitative comparison of flowering phenology traits among trees, perennial herbs, and annuals in a temperate plant community
Source: Am J Bot. 2019 Nov 14;106(12):1545–57. doi: 10.1002/ajb2.1387 (PMC6973048; doi:10.1002/ajb2.1387)
Supplement: Supplementary file 6 — APPENDIX S6. Model selection using Bayesian information criterion (BIC). [file AJB2-106-1545-s006.docx]

**Appendix S6.** **Model selection using Bayesian information criterion (BIC).**

Bold numbers are the smallest values for each phenological variable. Total flowering length of species (TFL), mean flowering length of individuals (MFL) and its variance (VFL).

| Phenological variables | Linear | | Quadratic | | Logarithmic | | Logistic | |
| --- | --- | --- | --- | --- | --- | --- | --- | --- |
|  | Df | BIC | Df | BIC | Df | BIC | Df | BIC |
| TFL | 3 | -1312.1 | 4 | -1498.7 | 3 | -1765.8 | 4 | **-2078.1** |
| MFL | 3 | -5964.1 | 4 | -5962.8 | 3 | **-5969.1** | 4 | -5965.9 |
| VFL | 3 | -3866.0 | 4 | -3866.2 | 3 | **-3867.0** | 4 | -3861.5 |
| Variance of onset date | 3 | -3843.5 | 4 | -3836.7 | 3 | **-3843.8** | 4 | -3836.7 |
| Skewness | 3 | 1642.0 | 4 | 1585.4 | 3 | 1569.5 | 4 | **1562.8** |
| Kurtosis | 3 | -1735.0 | 4 | -1890.7 | 3 | -2083.1 | 4 | **-2263.5** |
| *Iδ* | 3 | -23499.2 | 4 | **-24245.2** | 3 | -4053.4 | 4 | -14300.2 |
