# Supplementary material for: Quantitative comparison of flowering phenology traits among trees, perennial herbs, and annuals in a temperate plant community
Source: Am J Bot. 2019 Nov 14;106(12):1545–57. doi: 10.1002/ajb2.1387 (PMC6973048; doi:10.1002/ajb2.1387)
Supplement: Supplementary file 7 — APPENDIX S7. Tests of phylogenetic signals. [file AJB2-106-1545-s007.docx]

**Appendix S7.** Tests of phylogenetic signals.

| Dataset | Raw data | | n = 5 | | n = 7 | | n = 12 | | n = 18 | | n = 22 | |
| --- | --- | --- | --- | --- | --- | --- | --- | --- | --- | --- | --- | --- |
| Phenological  variables | *K* | *P*-value | *K* | *P*-value | *K* | *P*-value | *K* | *P*-value | *K* | *P*-value | *K* | *P*-value |
| TFL | 0.242 | 0.155 | 0.238 | 0.174 | 0.236 | 0.176 | 0.235 | 0.219 | 0.229 | 0.217 | 0.229 | 0.201 |
| MFL | 0.114 | 0.903 | 0.204 | 0.352 | 0.204 | 0.358 | 0.204 | 0.334 | 0.204 | 0.366 | 0.204 | 0.331 |
| VFL | 0.159 | 0.553 | 0.232 | 0.335 | 0.231 | 0.372 | 0.229 | 0.393 | 0.228 | 0.388 | 0.227 | 0.429 |
| Variance of  onset date | 0.140 | 0.640 | 0.238 | 0.315 | 0.238 | 0.300 | 0.239 | 0.311 | 0.239 | 0.319 | 0.240 | 0.282 |
| Skewness | 0.163 | 0.557 | 0.276 | 0.086 | 0.223 | 0.190 | 0.219 | 0.246 | 0.209 | 0.317 | 0.206 | 0.298 |
| Kurtosis | 0.191 | 0.413 | 0.179 | 0.486 | 0.179 | 0.541 | 0.163 | 0.625 | 0.185 | 0.561 | 0.175 | 0.601 |
| *Iδ* | 0.362 | 0.106 | 0.239 | 0.236 | 0.248 | 0.216 | 0.256 | 0.185 | 0.259 | 0.175 | 0.229 | 0.234 |
