# Supplementary material for: Quantitative comparison of flowering phenology traits among trees, perennial herbs, and annuals in a temperate plant community
Source: Am J Bot. 2019 Nov 14;106(12):1545–57. doi: 10.1002/ajb2.1387 (PMC6973048; doi:10.1002/ajb2.1387)
Supplement: Supplementary file 8 — APPENDIX S8. GLMMs examining the effects of year and the number of observed individuals on phenological variables. [file AJB2-106-1545-s008.docx]

**Appendix S8. GLMMs examining the effects of year and the number of observed individuals on phenological variables.**

GLMMs examining the effects of year and the number of observed individuals on phenological variables including total flowering length of species (TFL), mean flowering length of individuals (MFL) and its variance (VFL). * *p* < 0.05, *** *p* < 0.005.

| **Phenological variables** |  | **Explanatory variables** | | | | | | | | | |
| --- | --- | --- | --- | --- | --- | --- | --- | --- | --- | --- | --- |
|  |  | **Intercept** | | **Year** | | | **Number of observed individuals** | | | | |
|  |  |  |  | **Slope** | ***P*-value** |  |  | **Slope** | ***P*-value** |  |  |
| TFL |  | 3.50 |  | -0.02 | 0.75 |  |  | 0.02 | 0.00 | *** |  |
| MFL |  | 2.81 |  | -0.12 | 0.20 |  |  | 0.01 | 0.03 | * |  |
| VFL |  | 4.42 |  | -0.38 | 0.10 |  |  | 0.02 | 0.11 |  |  |
| Variance of onset date |  | 3.71 |  | 0.70 | 0.02 | * |  | 0.00 | 1.00 |  |  |
| Skewness |  | - |  | - | - |  |  | - | - |  |  |
| Kurtosis |  | 1.01 |  | -0.17 | 0.06 |  |  | 0.00 | 0.50 |  |  |
| *Iδ* |  | 1.86 |  | 0.08 | 0.40 |  |  | -0.02 | 0.04 | * |  |
