# Supplementary material for: Quantitative comparison of flowering phenology traits among trees, perennial herbs, and annuals in a temperate plant community
Source: Am J Bot. 2019 Nov 14;106(12):1545–57. doi: 10.1002/ajb2.1387 (PMC6973048; doi:10.1002/ajb2.1387)
Supplement: Supplementary file 9 — APPENDIX S9. LMM examining the effects of year and the number of observed individuals on skewness. [file AJB2-106-1545-s009.docx]

**Appendix S9. LMM examining the effects of year and the number of observed individuals on skewness.** ** *P* < 0.01.

| Model | *χ*^2^ | **df** | ***P*** |  |
| --- | --- | --- | --- | --- |
| 1 | 9.34 | 1 | 0.00 | ** |
| 2 | 0.00 | 0 | 1.00 |  |
