# Supplementary material for: Quantitative comparison of flowering phenology traits among trees, perennial herbs, and annuals in a temperate plant community
Source: Am J Bot. 2019 Nov 14;106(12):1545–57. doi: 10.1002/ajb2.1387 (PMC6973048; doi:10.1002/ajb2.1387)
Supplement: Supplementary file 10 — APPENDIX S10. Tests among life forms for each phenological variable. [file AJB2-106-1545-s010.docx]

Appendix S10. Tests among life forms for each phenological variable for 5, 7, 18, and 22 individuals, and the raw data. Student’s *t* test, Wilcoxon rank sum test, or Fligner-Policello test was used depending on normality and variance of groups. *P*-values were adjusted using the Holm method. * *P* < 0.05, ** *P* < 0.01, *** *P* < 0.005.

| **Dataset** | | ***n* = 5** | | | ***n* = 7** | | | ***n* = 18** | | | ***n* = 22** | | |
| --- | --- | --- | --- | --- | --- | --- | --- | --- | --- | --- | --- | --- | --- |
| **Variable** | **Pairs** | **Test statistic** | ***P*** |  | **Test statistic** | ***P*** |  | **Test statistic** | ***P*** |  | **Test statistic** | ***P*** |  |
| TFL | Tree–Perennial | 60.00 | 0.177 |  | 63.00 | 0.237 |  | 66.00 | 0.311 |  | 66.00 | 0.311 |  |
|  | Perennial–Annual | 121.00 | 0.347 |  | 120.00 | 0.330 |  | 112.00 | 0.311 |  | 111.00 | 0.311 |  |
|  | Annual–Tree | 2.86 | 0.022 | * | 2.97 | 0.017 | * | 3.03 | 0.015 | * | 3.06 | 0.013 | * |
| MFL | Tree–Perennial | -1.58 | 0.251 |  | -1.58 | 0.253 |  | -1.56 | 0.262 |  | -1.56 | 0.264 |  |
|  | Perennial–Annual | 0.07 | 0.948 |  | 0.07 | 0.946 |  | 0.07 | 0.943 |  | 0.07 | 0.942 |  |
|  | Annual–Tree | 2.21 | 0.105 |  | 2.20 | 0.106 |  | 2.18 | 0.111 |  | 2.18 | 0.112 |  |
| VFL | Tree–Perennial | -1.89 | 0.117 |  | -1.89 | 0.117 |  | -1.89 | 0.117 |  | -1.84 | 0.131 |  |
|  | Perennial–Annual | -0.71 | 0.481 |  | -0.67 | 0.500 |  | -0.71 | 0.479 |  | -0.71 | 0.479 |  |
|  | Annual–Tree | 200.00 | 0.027 | * | 200.00 | 0.027 | * | 199.00 | 0.030 | * | 199.00 | 0.030 | * |
| Variance of onset date | Tree–Perennial | -1.73 | 0.250 |  | -1.73 | 0.250 |  | -1.73 | 0.250 |  | -1.73 | 0.250 |  |
|  | Perennial–Annual | 0.41 | 0.683 |  | 0.44 | 0.661 |  | 0.44 | 0.661 |  | 0.44 | 0.661 |  |
|  | Annual–Tree | 1.06 | 0.581 |  | 1.10 | 0.547 |  | 1.22 | 0.448 |  | 1.30 | 0.390 |  |
| Skewness | Tree–Perennial | -0.26 | 1.000 |  | -0.71 | 0.971 |  | 87.00 | 0.988 |  | 87.00 | 1.000 |  |
|  | Perennial–Annual | -0.30 | 1.000 |  | -0.19 | 0.971 |  | -0.69 | 0.988 |  | -0.66 | 1.000 |  |
|  | Annual–Tree | 0.94 | 1.000 |  | 1.27 | 0.640 |  | 174.00 | 0.329 |  | 177.00 | 0.260 |  |
| Kurtosis | Tree–Perennial | -0.90 | 0.752 |  | 69.00 | 0.401 |  | -1.33 | 0.367 |  | -1.28 | 0.400 |  |
|  | Perennial–Annual | -0.17 | 0.868 |  | -0.51 | 0.615 |  | -1.05 | 0.367 |  | -1.18 | 0.400 |  |
|  | Annual–Tree | 1.22 | 0.691 |  | 179.00 | 0.221 |  | 2.01 | 0.135 |  | 1.95 | 0.153 |  |
| *Iδ* | Tree–Perennial | 1.92 | 0.132 |  | 146.00 | 0.066 |  | 149.00 | 0.048 | * | 150.00 | 0.043 | * |
|  | Perennial–Annual | 146.00 | 0.908 |  | 141.00 | 0.780 |  | 132.00 | 0.564 |  | 132.00 | 0.564 |  |
|  | Annual–Tree | 73.00 | 0.108 |  | 68.00 | 0.066 |  | 65.00 | 0.048 | * | 64.00 | 0.043 | * |
