# Supplementary material for: Quantitative comparison of flowering phenology traits among trees, perennial herbs, and annuals in a temperate plant community
Source: Am J Bot. 2019 Nov 14;106(12):1545–57. doi: 10.1002/ajb2.1387 (PMC6973048; doi:10.1002/ajb2.1387)
Supplement: Supplementary file 11 — APPENDIX S11. The results of comparing each phenological variable for the species among life forms in the case of raw data. [file AJB2-106-1545-s011.docx]

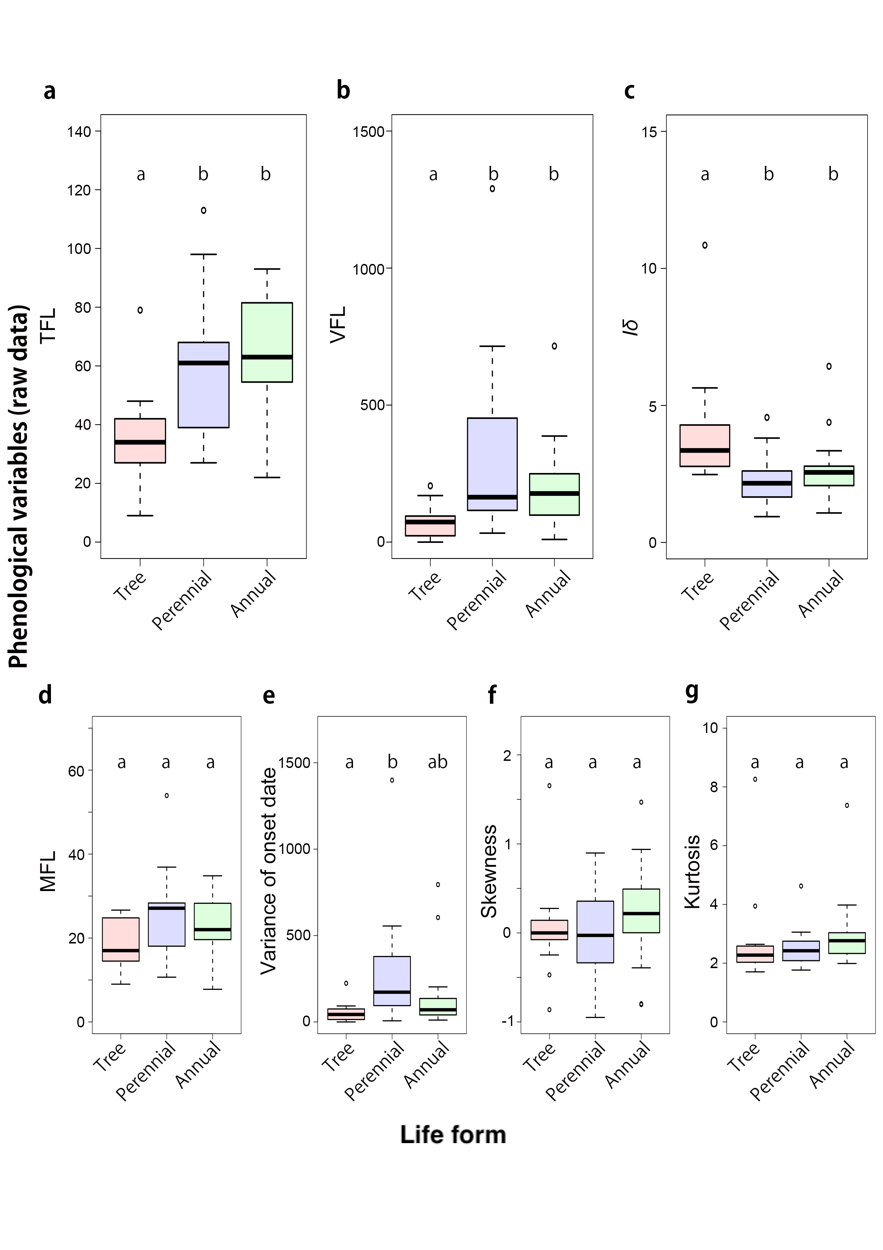


**Appendix S11. The results of comparing raw data for each phenological variable for the species among life forms.**

(a) TFL: total flowering length, (b) VFL: variance of mean flowering length, (c) *Iδ*, (d) MFL: mean flowering length, (e) variance of onset dates, (f) skewness, and (g) kurtosis of tree (red box; *n* = 13), perennial herbs (blue box; *n* = 15), and annual herbs (green box; *n* = 20) are shown. The black line inside the box shows the median, the box shows the first quartile to the third quartile, the upper and lower lines show the maximum and minimum values in the range of 1.5 times the length of the box, and the white circles show the outliers. The letters above the boxes indicate significance; different letters indicate a significant difference.
