# Supplementary material for: Quantitative comparison of flowering phenology traits among trees, perennial herbs, and annuals in a temperate plant community
Source: Am J Bot. 2019 Nov 14;106(12):1545–57. doi: 10.1002/ajb2.1387 (PMC6973048; doi:10.1002/ajb2.1387)
Supplement: Supplementary file 12 — APPENDIX S12. Distributions of onset date in trees, perennial herbs, and annual herbs. [file AJB2-106-1545-s012.docx]

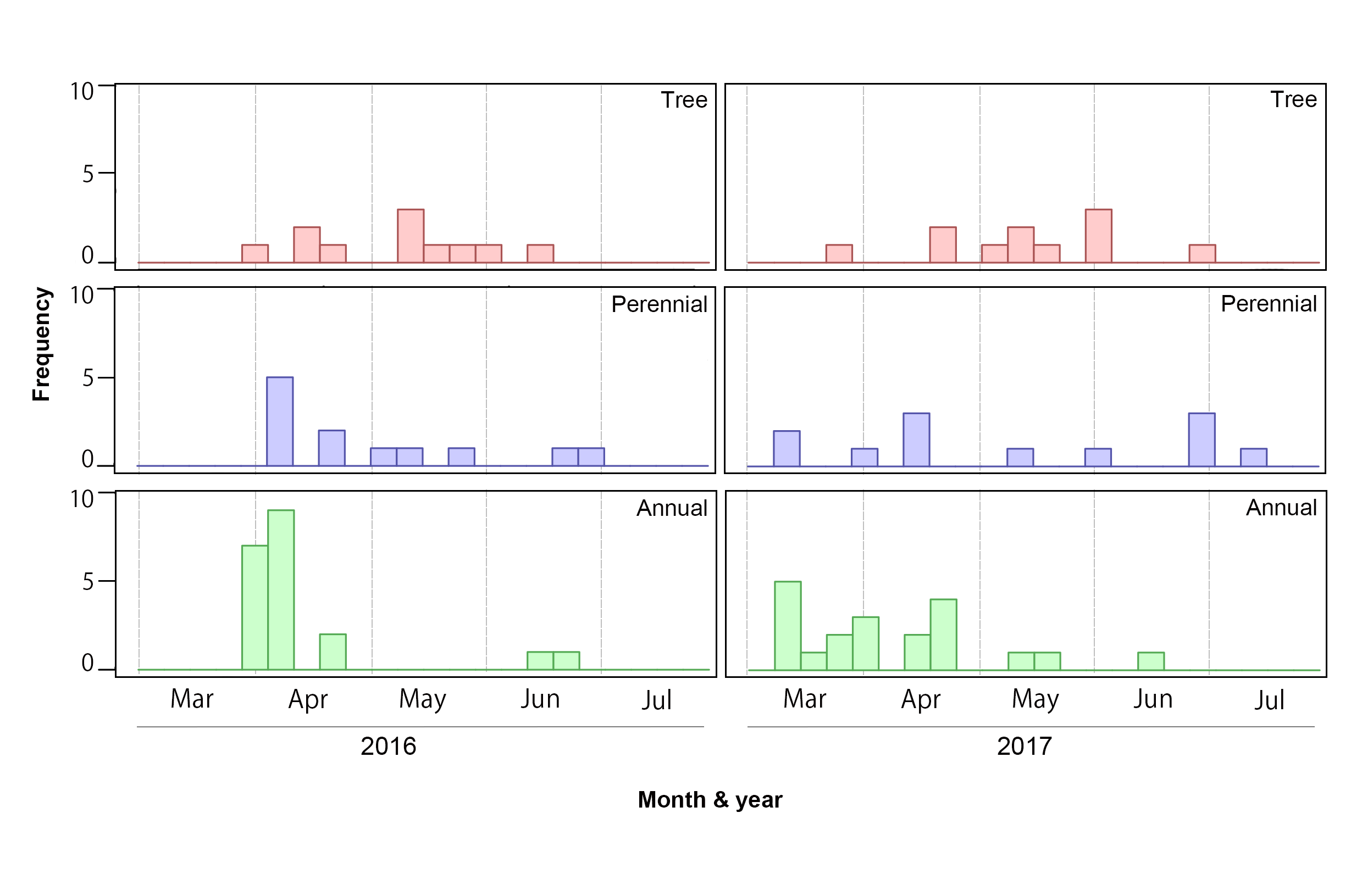


**Appendix S12. Distributions of flowering onset date for trees (red), perennial herbs (blue), and annual herbs in 2016 (left), and in 2017 (right).** Vertical axis shows number of species that started to flower.
