# Supplementary material for: Quantitative comparison of flowering phenology traits among trees, perennial herbs, and annuals in a temperate plant community
Source: Am J Bot. 2019 Nov 14;106(12):1545–57. doi: 10.1002/ajb2.1387 (PMC6973048; doi:10.1002/ajb2.1387)
Supplement: Supplementary file 13 — APPENDIX S13. Differences in the distributions of onset date from normal. [file AJB2-106-1545-s013.docx]

**Appendix S13.** **Differences in the distributions of onset date from normal.**

|  | 2016 | | | 2017 | | |
| --- | --- | --- | --- | --- | --- | --- |
| Life form | Skewness | *z* | *P* | Skewness | *z* | *P* |
| Tree | -0.06 | -0.11 | 0.910 | -0.27 | -0.50 | 0.615 |
| Perennial | 0.91 | 1.65 | 0.098 | 0.14 | 0.26 | 0.798 |
| Annual | 2.45 | 4.04 | 0.000 | 0.80 | 1.71 | 0.088 |
